# Alternative splicing of *Clock* transcript mediates the response of circadian clocks to temperature changes

**DOI:** 10.1073/pnas.2410680121

**Published:** 2024-12-04

**Authors:** Yao D. Cai, Xianhui Liu, Gary K. Chow, Sergio Hidalgo, Kiya C. Jackson, Cameron D. Vasquez, Zita Y. Gao, Vu H. Lam, Christine A. Tabuloc, Haiyan Zheng, Caifeng Zhao, Joanna C. Chiu

**Affiliations:** ^a^Department of Entomology and Nematology, College of Agricultural and Environmental Sciences, University of California Davis, Davis, CA 95616; ^b^Cambridge-Suda Genomic Resource Center, Suzhou Medical College, Soochow University, Suzhou, Jiangsu 215123, China; ^c^Biological Mass Spectrometry Facility, Center for Advanced Biotechnology and Medicine, Robert Wood Johnson Medical School and Rutgers, The State University of New Jersey, Piscataway, NJ 08854

**Keywords:** temperature, alternative splicing, phosphorylation, post-translational modification, circadian rhythm

## Abstract

Circadian clocks respond to temperature changes over the calendar year, allowing organisms to adjust their daily biological rhythms to optimize health and fitness. In *Drosophila*, seasonal adaptations are regulated by temperature-sensitive alternative splicing (AS) of *period* (*per*) and *timeless* (*tim*) genes that encode key transcriptional repressors of clock gene expression. Although *Clock* (*Clk*) gene encodes the critical activator of circadian gene expression, AS of its transcripts and its potential role in temperature regulation of clock function have not been explored. Here, we observed that *Clk* transcripts undergo temperature-sensitive AS. Specifically, cold temperature leads to the production of an alternative *Clk* transcript, hereinafter termed *Clk*-cold, which encodes a CLK isoform with an in-frame deletion of four amino acids proximal to the DNA binding domain. Notably, serine 13 (S13), which we found to be a CK1α-dependent phosphorylation site, is deleted in CLK-cold protein. We demonstrated that upon phosphorylation at CLK(S13), CLK-DNA interaction is reduced, thus decreasing transcriptional activity of CLK. This is in agreement with our findings that CLK occupancy at clock genes and transcriptional output are elevated at cold temperature likely due to higher amounts of CLK-cold isoforms that lack S13 residue. Finally, we showed that PER promotes CK1α-dependent phosphorylation of CLK(S13), supporting kinase-scaffolding role of repressor proteins as a conserved feature in the regulation of eukaryotic circadian clocks. This study provides insights into the complex collaboration between AS and phospho-regulation in shaping temperature responses of the circadian clock.

## Significance

Adaptation to environmental temperature changes is critical for the survival of most organisms. Circadian clocks respond to temperature to modulate daily biological rhythms over the calendar year, yet our understanding of the mechanisms underlying this phenomenon is far from complete. Here, we found that in flies, the *Clock* (*Clk*) gene exhibits temperature-sensitive alternative splicing to regulate the transcription of CLOCK target genes and modulate behavioral rhythmicity at different temperatures. Among the deleted amino acid residues in CLOCK-cold, the prevalent protein isoform at low temperature, we identified a phosphorylation site that regulates CLOCK transcriptional activity by modulating CLOCK-DNA binding. This study provides insights into the complex collaboration between alternative splicing and phospho-regulation in shaping temperature responses of the circadian clock.

Circadian clocks regulate daily physiological and behavioral rhythms to optimize health and fitness in organisms from all domains of life (1–7). In animals and other eukaryotes, the pace of the circadian clock is highly responsive to environmental cues, with light–dark cycles as the strongest and most-studied time cue to entrain and reset circadian clocks (8). Temperature is another important environmental cue that greatly affects the physiology of animals. Although circadian clocks can maintain their pace over a wide range of temperatures, a phenomenon called temperature compensation (9), circadian clocks can also adapt to more extreme temperatures. For example, under cold conditions, the expression of *Period2*, a key component in the molecular clock, is induced in brown adipose tissue of mice to increase systemic heat production and resistance to cold temperature (10). At present, there are still significant knowledge gaps regarding the molecular mechanisms by which circadian clocks respond and adapt to cold temperatures to optimize daily biological rhythms.

A number of studies have established alternative splicing (AS) as an important mechanism that mediate temperature responses of circadian clocks in animals (11–13), plants (14), and fungi (15–17). The molecular clocks in most organisms are composed of transcription–translation feedback loops (TTFLs) (2, 5) that regulate daily rhythmicity in clock gene expression. The TTFL model in animal clocks was first formulated in *Drosophila* in 1990 (18). It is composed of positive elements, CLOCK (CLK) and CYCLE (CYC), that drive the transcription of clock genes including genes encoding the negative elements, PERIOD (PER) and TIMELESS (TIM), which feedback to repress the activity of positive elements. The next daily cycle of transcription begins as repression is relieved by proteasomal degradation of PER and TIM.

There has been great progress in understanding the temperature-sensitive AS of negative elements in *Drosophila* clocks, in parallel to that in fungal (15–17) and plant clocks (14, 19, 20). AS of *per* and *tim* improves fitness of flies by minimizing locomotor activity during the time with adverse environmental temperature, such as cold night and hot midday (13, 21, 22). Cold-induced AS of *per dmpi8* intron at the 3′ UTR region (13) promotes advanced evening locomotor activity peak by stimulating transcription of *daywake* (23). In addition to *per*, four splice variants of *tim* are found to be differentially expressed in response to temperature changes (21, 24, 25). Constitutively spliced *tim*-L (full-length isoform) is dominant at moderate temperature (25 °C) (25). In colder temperatures (10 to 18 °C), increased expression of *tim*-cold and *tim*-short and cold (*tim*-sc) are linked to advanced evening locomotor activity peak. Increased expression of *tim*-M in warmer temperatures (29 to 35 °C) contributes to the delay of evening behavioral peak (21). However, whether transcripts of the positive elements in *Drosophila* clocks undergo temperature-sensitive AS remains unexplored. This is surprising given CLK, in partner with CYC (homolog of mammalian BMAL1), are the major transcriptional activators of clock gene expression and are critical for driving clock output (26, 27).

In comparison to the paucity of studies that explore the regulation of CLK function by AS, there have been experimental evidence and mathematical modeling that demonstrated the importance of posttranslational regulation of CLK activity. In particular, phosphorylation plays key roles in shaping the daily oscillation of CLK transcriptional activity, while CLK protein abundance stays constant over the 24-h day-night cycle (28–30). In midday to early evening, newly synthesized hypophosphorylated CLK binds DNA and exhibits high transcriptional activity after nuclear entry (28, 31). Due to PER-dependent recruitment of kinases, highly phosphorylated CLK appearing at late night exhibits downswing of transcriptional activity and is gradually removed from circadian promoters (28, 32, 33). Dephosphorylation of CLK that occurs in early day replenishes the pool of transcriptionally active hypophosphorylated CLK (30). However, it is unknown how the daily CLK phosphorylation profile and transcriptional activity change with temperature. Furthermore, whether posttranscriptional mechanism such as AS modulates daily CLK phosphorylation profile to regulate cold adaptation of molecular clocks remains a significant gap in knowledge.

Here, we observed that cold temperature increases the usage of an alternative 3′ splice site in *Clk* exon 2, leading to the production of an alternative transcript that encodes a CLK protein missing four amino acids proximal to the N-terminal DNA basic helix–loop–helix (bHLH) binding domain. We named this shorter transcript hereinafter *Clk*-cold. We demonstrated that CLK-cold protein exhibits increased occupancy at CLK target gene promoters and elevates CLK target gene expression at cold temperature. Among the four amino acids deleted in CLK-cold protein, we used mass spectrometry proteomics and phosphospecific antibodies to show that CLK(S13) is phosphorylated by casein kinase 1α (CK1α), primarily during the early part of the daily cycle. By analyzing the behavioral and molecular output of *Clk*(S13D) phosphomimetic mutants, we provided evidence supporting the function of CLK(S13) phosphorylation in modulating CLK occupancy at CLK target gene promoters. This maintains daily CLK target mRNA expression and PDF neuropeptide accumulation rhythm to drive rhythmic locomotor activities. Finally, we observed that disrupting CLK(S13) phosphorylation in vivo compromises circadian locomotor activity rhythms at colder temperature. Based upon these findings and given that CLK-cold lacks CLK(S13) phospho-regulation, we propose a model describing a mechanism by which AS of *Clk* transcript mediates the response of circadian clocks to temperature changes by modulating transcriptional activity of CLK via CK1α-dependent phosphorylation.

## Results

### Temperature Regulates Alternative 3’ Splice Site Selection in Exon 2 of *Clk*.

**Fig. 1.**
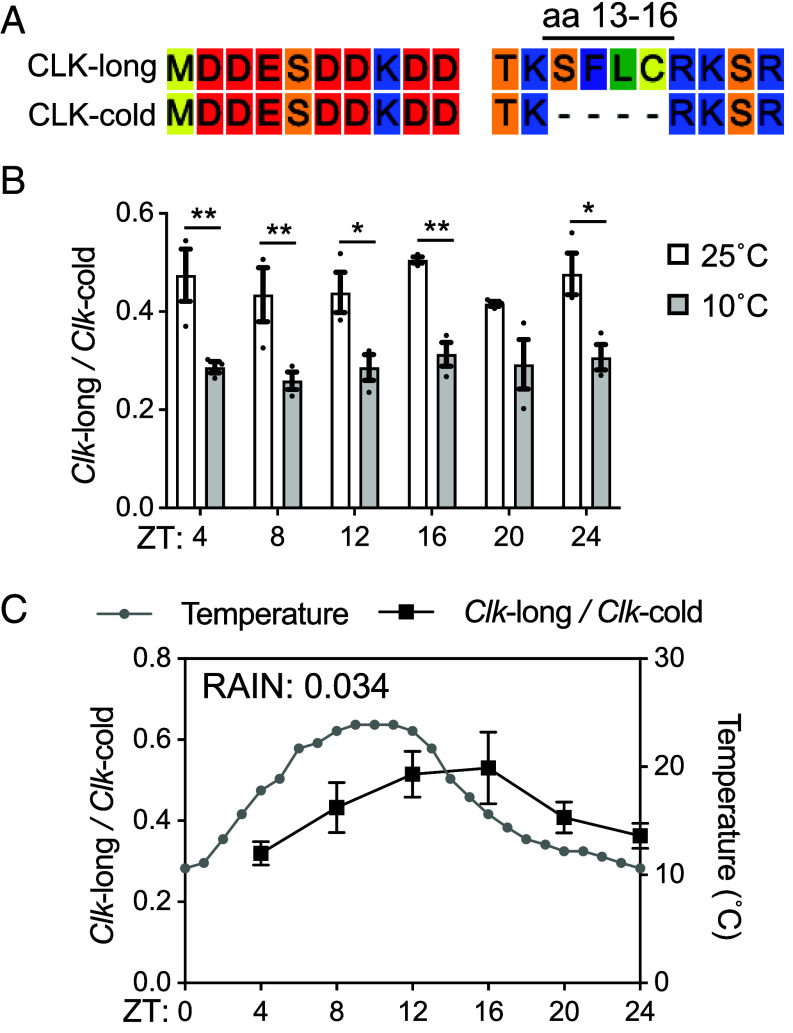
Cold induces alternative splicing of *Clk*. (*A*) Alignment of amino acid sequences encoded by two *Clk* transcripts isolated from heads of *w*^1118^ flies. Four amino acids (aa 13-16) are absent in CLK-cold. (*B* and *C*) The ratio of *Clk*-long to *Clk*-cold was measured in heads of *w*^1118^ flies by quantitative RT-PCR. Flies were entrained in 12 h:12 h LD at indicated constant temperature in (*B*) and 14 h:10 h LD at natural daily temperature cycles in (*C*). Flies were collected on LD3 at indicated time-points (ZT). Error bars indicate ± SEM (n = 3), **P < 0.01, *P < 0.05, two-way ANOVA and Šídák’s post hoc test.

We first sought to determine whether *Clk* transcripts exhibit alternative splicing, as in the case of other key clock genes such as *per* and *tim*. When *Drosophila Clk* transcripts were first cloned in 1998 by three independent labs, two different cDNA products were identified (34–36). The longer transcript, which we termed *Clk-*long, encodes the canonical CLK protein. The other slightly shorter transcript, hereinafter termed *Clk*-cold, encodes a CLK protein with a four amino acid (aa) deletion at aa13-16 (Fig. 1*A*). The annotation of the genomic *Clk* sequence revealed a potential alternative 3’ splice site in exon 2 of *Clk*, which may have produced the two cDNA of different lengths. Indeed, both transcripts are expressed in fly heads, according to deep sequencing of circadian transcriptome in Hughes et al. (37). Cell-specific RNA-seq data from Wang et al. (38) also indicated that both *Clk-*long and *Clk-*cold are expressed in several circadian neuronal cell types, including small lateral ventral neurons (sLN_v_), the key pacemaker neurons (*SI Appendix*, Fig. S1*A*). Given that the four aa deletion of CLK-cold is adjacent to the bHLH DNA-binding domain (aa17-62) (35, 36), we hypothesize that differential expression of these two transcripts could impact the function of the molecular clock.

To further confirm the expression of both *Clk-*long and *Clk-*cold, we generated *Clk* cDNA fragments that span exons 1 and 2 from RNA extracted from whole heads of *w*^1118^ flies collected at a time-point when *Clk* mRNA expression is high (35) (*SI Appendix*, Fig. S1*B*). We noticed three potentially different cDNAs when we analyzed the amplified cDNAs on agarose gel (*SI Appendix*, Fig. S1*C*). Sanger sequencing revealed that the bottom band represents *Clk*-cold and the middle band represents *Clk*-long (*SI Appendix*, Fig. S1*D*). The top band represents a hybrid of *Clk*-cold and *Clk*-long, potentially an PCR artifact that exhibits slower mobility on the agarose gel. These data support published sequencing data (37, 38) reporting *Clk*-long and *Clk*-cold are expressed in fly heads.

To determine whether AS of *Clk* is temperature-sensitive, we evaluated daily relative abundance of *Clk*-long and *Clk*-cold from head extracts of *w*^1118^ flies entrained under LD at 25 and 10 °C, respectively (Fig. 1*B*). 10 °C was chosen to better simulate a more naturalistic cold temperature, e.g., morning of a spring day, during which *Drosophila* flies are viable and active (39). Nested qPCR assays targeting the alternative 3′ splice site region allow quantitative analysis of both transcripts (*SI Appendix*, Fig. S1*E*). At 25 °C, *Clk-*long and *Clk-*cold were expressed at 1:2 ratio. However, the relative abundance of *Clk*-long decreases and *Clk*-cold becomes even more prevalent at 10 °C. AS of *Clk* did not exhibit daily oscillation at constant temperature, given the ratio does not oscillate under LD condition, as determined by rhythmicity analysis incorporating nonparametric methods (RAIN) (40) (25 °C: *P* = 0.81; 10 °C: *P* = 0.86, RAIN).

Even in endothermic organisms such as mice, body temperature rhythm was found to drive rhythmic AS of over 1,000 exons (12). We therefore hypothesize that environmental temperature cycles drive rhythmic AS of *Clk* in ectothermic *Drosophila*. We measured expression of *Clk-*long and *Clk-*cold under semi-natural conditions, where the incubators were set to mimic a typical day in May in Davis, CA (weatherspark.com), with diurnal temperatures ranging from 10 to 25 °C (Fig. 1*C*). We observed steady increase of relative *Clk*-long in response to increasing temperature in the first half of the day. As temperature decreases in the second half of the day, relative level of *Clk*-long decreases. Under environmental temperature cycles, AS of *Clk* is rhythmic (*P* = 0.034, RAIN). These data suggest that AS of *Clk* is sensitive to environmental temperature changes.

### Elevated CLK-DNA Binding Contributes to Increased CLK Target mRNA Level at Cold Temperature.

**Fig. 2.**
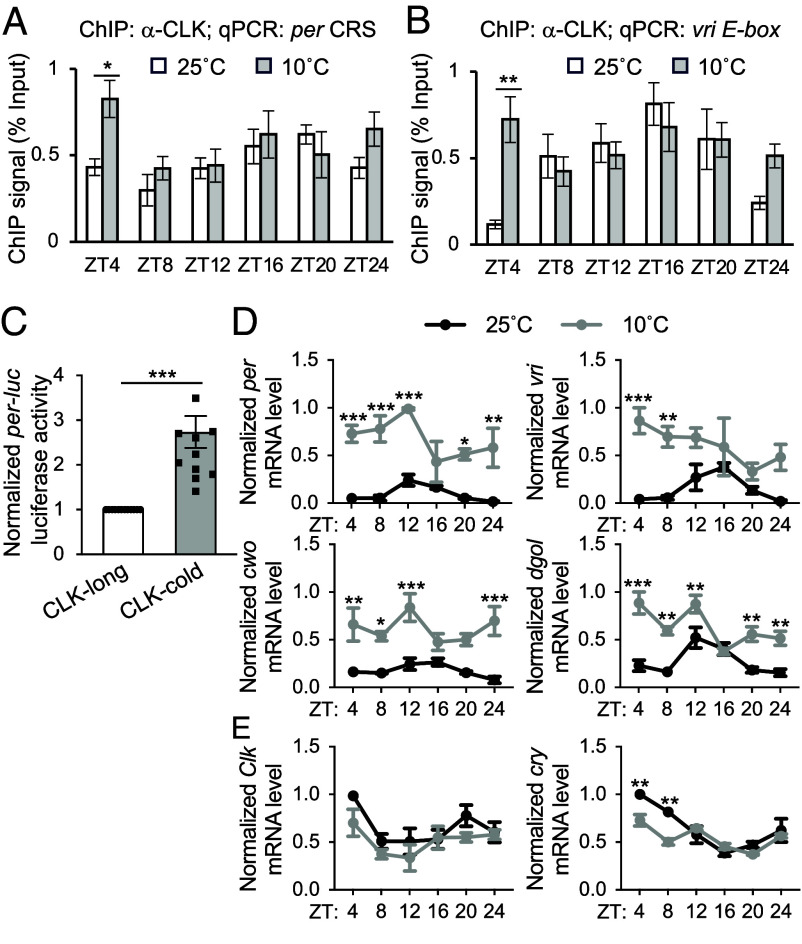
Elevated CLK-DNA binding contributes to increased mRNA level of CLK targets at low temperature. (*A* and *B*) ChIP assays using fly head extracts comparing daily CLK occupancy at the *per* and *vri* promoter in *w*^1118^ flies collected at 25 and 10 °C. CLK-ChIP signals were normalized to % input. ChIP signals for an intergenic region were used for nonspecific background deduction. Flies were entrained in 12 h:12 h LD and collected on LD3 at indicated time-points (ZT) (n = 5). Error bars indicate ± SEM, **P < 0.01, *P < 0.05, two-way ANOVA and Šídák’s post hoc test. (*C*) *per-E-box-luciferase* (*per-luc*) reporter assay performed in *Drosophila* S2 cells. Luciferase activity was normalized to CLK-long and expressed as fold change relative to CLK-long. Error bars indicate ± SEM (n = 12), ***P < 0.001, two-tailed Student’s *t* test. (*D* and *E*) Steady state daily mRNA expression of CLK targets (*per*, *vri*, *cwo,* and *dgol*) and non-CLK targets (*Clk* and *cry*) in heads of *w*^1118^ flies. Flies were entrained in 12 h:12 h LD and collected on LD3 at indicated temperatures and time-points (ZT) (n = 3). Error bars indicate ± SEM, ***P < 0.001, **P < 0.01, *P < 0.05, two-way ANOVA and Šídák’s post hoc test.

Since CLK-cold is missing four amino acids (aa 13-16) adjacent to the N-terminal bHLH DNA binding domain (aa17-62), we hypothesize that CLK-cold displays altered CLK-DNA binding activity. To test this hypothesis, we performed CLK chromatin immunoprecipitation (CLK-ChIP) followed by qPCR using extracts from adult fly heads collected at 25 °C vs. 10 °C. In agreement with published data (41), we observed rhythmic CLK occupancy at the *per* CRS, a region within the promoter critical for generating rhythmic *per* expression (42) and at the *vri E-box* (CLK binding motif) at 25 °C (Fig. 2 *A* and *B*) (*per* CRS: *P* = 0.014; *vri E-box*: *P* = 0.002, RAIN). However, we observed dampening of daily rhythmicity of CLK occupancy at 10 °C (*per* CRS: *P* = 0.582; *vri E-box*: *P* = 0.142, RAIN), likely due to significantly higher CLK occupancy at both circadian promoters at ZT4. Our ChIP data suggest CLK-cold exhibits elevated DNA binding activity as compared to CLK-long, most notably at ZT4.

We next asked whether altered DNA binding activity regulates transcriptional activity of each CLK isoforms. We first assayed transcriptional activity of CLK in *Drosophila* S2 cells using a *per*-*luc* reporter assay (36). We compared *per-luc* reporter gene activity in S2 cells expressing CLK-long or CLK-cold (Fig. 2*C*). The 2.7-fold increased reporter gene activity in CLK-cold suggests a significantly elevated transcriptional activity of CLK-cold, as compared to CLK-long. Transcriptional activity of CLK-cold was also inferred by measuring expression of known CLK target genes in flies entrained in 12 h:12 h LD at 25 °C vs. 10 °C, respectively (Fig. 2*D*). At 10 °C where CLK-cold is elevated, mRNA levels of CLK targets, including *per*, *vrille (vri)*, *clockwork orange (cwo)*, *goliath* (*dgol)*, are significantly higher than the levels observed at 25 °C at multiple time-points. Similarly, total *tim* and *pdp1* mRNA levels are elevated at 10 °C, despite the fact that these two genes have temperature-sensitive AS (*SI Appendix*, Fig. S2). In contrast, mRNA levels of non-CLK targets including *Clk* and *cryptochrome* (*cry*) did not increase at 10 °C (Fig. 2*E*). This strongly indicates that the observed elevated expression at cold temperature is not a general phenomenon. Taken together, our results revealed that cold-induced AS of *Clk* results in a CLK protein that binds more readily to DNA at an early part of the day-night cycle, thereby increasing expression of CLK target genes.

### CLK(S13) Is a CK1α-Dependent Phosphorylation Site That Regulates Transcriptional Activity of CLK.

We next sought to explain how the four-aa deletion in CLK-cold alters CLK transcriptional output. Since CLK phosphorylation status displays daily rhythmicity and is tightly correlated with its transcriptional activity (28, 30, 43), we investigated whether these four amino acids overlap with any kinase-targeted motif. Kinase prediction using group-based phosphorylation site predicting and scoring (GPS) 5.0 (44) showed that the serine 13 (S13) residue could be a potential substrate of kinases from several kinase families (*SI Appendix*, Table S1). Previous studies suggested that NEMO (45), SGG (46–48), CK2 (49), and CK1α (50) could be CLK kinases and CLK kinases could be recruited by PER to phosphorylate CLK. Among these kinases, GPS 5.0 kinase prediction algorithm identified CK1α to be the most likely candidate to phosphorylate CLK(S13).

**Fig. 3.**
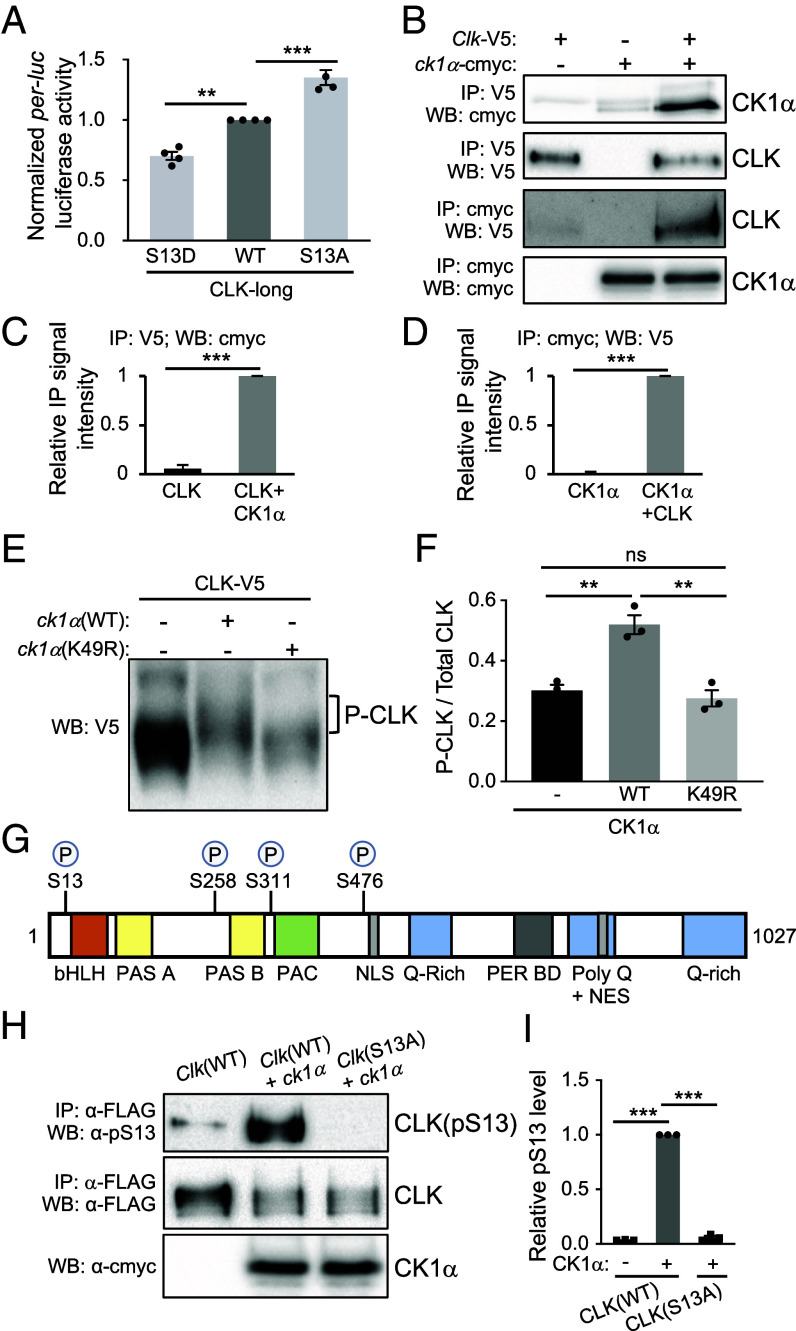
CLK(S13) is a substrate of CK1α. (*A*) *per-E-box-luciferase* (*per-luc*) reporter assay performed in *Drosophila* S2 cells. Luciferase activity was normalized to CLK(WT) and expressed as fold change relative to CLK-long. Error bars indicate ± SEM (n = 4), ***P < 0.001, one-way ANOVA and Dunnett post hoc test. (*B*) Western blots showing reciprocal coIPs to examine the interactions of CLK and CK1α. S2 cells were cotransfected with 0.8 μg of pAc-*Clk*-V5-His and 0.8 μg of pMT-*ck1α*-6xc-myc or transfected with a single plasmid for control experiments. Protein extracts were divided into two equal aliquots, and each aliquot was independently incubated with either α-c-myc beads or α-V5 beads. Immuno-complexes were analyzed by western blotting in the presence of the indicated antibody. (*C* and *D*) Bar graphs displaying quantification of reciprocal coIPs (*B*). Values for binding are normalized to amount of bait detected in the IPs and expressed as relative signal intensity (maximum value = 1). Error bars indicate ± SEM (n = 3), two-tailed Student’s *t* test. (*E*) Western blots showing mobility shift of CLK on a Phos-tag SDS-PAGE. S2 cells were transfected with 0.8 μg of pAc-*Clk*-V5 together with 0.6 μg of either pMT-*ck1α*-FH, pMT-*ck1*α(K49R)-FH, or pMT-FH. (*F*) Quantification of phosphorylated/total CLK in (*E*). Error bars indicate ± SEM (n = 3). ***P < 0.001, **P < 0.01, one-way ANOVA and Tukey’s post hoc test. (*G*) Schematic diagram depicting *Drosophila melanogaster* CLK (amino acid 1 to 1027) adapted from Mahesh et al. (51), and CK1α-dependent phosphorylation sites identified by mass spectrometry. Previously described domains of CLK: bHLH (aa 17-62) (35, 36); PAS-A (aa 96-144) (35, 36); PAS B (aa 264-309) (35, 36); C-terminal of PAS domain (PAC) (aa 315-379) (35); NLS (aa 480-494) (31); PER binding domain (PER BD) (aa 657-707) (52); Q-rich regions (aa 546-575, aa 957-1027), Poly-Q (aa 552-976) (35, 36) and NES (aa 840-864) (31). (*H*) *Drosophila* S2 cells were transfected with pAc-*Clk*(WT)-FLAG or pAc-*Clk*(S13A)-FLAG and cotransfected with an empty plasmid (pMT-cmyc-His) or pMT-*ck1*α-cmyc. Protein extracts were incubated with α-FLAG resin. Total CLK isoforms, CLK(pS13), and CK1α protein levels were analyzed by Western Blotting with the indicated antibodies. (*I*) Bar graph showing relative CLK pS13 levels in (*H*) normalized to total CLK isoforms. Error bars indicate ± SEM (n = 3), ***P < 0.001, one-way ANOVA and Dunnett post hoc test.

To first determine whether phosphorylation of S13 can regulate CLK transcriptional activity, we generated *Clk* plasmids expressing nonphosphorylatable S13 to Alanine (A) or phosphomimetic S13 to Aspartic Acid (D) mutations, both in the context of the CLK-long isoform, and tested their transcriptional activity using *per-luc* luciferase assays in *Drosophila* S2 cells. Whereas CLK(S13A) was found to exhibit higher transcriptional activity when compared to CLK-long (WT), CLK(S13D) had significantly lower transcriptional activity (Fig. 3*A*). Our results suggest S13 could indeed be a potential phosphorylation site that regulates CLK transcriptional activity. We then performed a series of experiments to determine whether CLK(S13) is a bona fide CK1α-dependent phosphorylation site. We first determined whether CLK interacts with CK1α by performing coimmunoprecipitation (coIP) assays using protein extracts from *Drosophila* S2 cells coexpressing CLK-V5 and CK1α-cmyc (Fig. 3 *B*–*D*). We detected interactions between CLK and CK1α when using CLK-V5 as bait (Fig. 3 *B* and *C*). Reciprocal coIP using CK1α-cmyc as bait resulted in the same conclusion (Fig. 3 *B* and *D*). Control experiments were performed using extracts of S2 cells expressing either of the proteins alone to demonstrate minimal nonspecific binding (Fig. 3 *B*–*D*).

Next, we determined whether CLK is phosphorylated by CK1α by assessing CLK mobility shift using sodium dodecyl sulfate polyacrylamide gel electrophoresis (SDS-PAGE). We analyzed CLK in protein extracts of *Drosophila* S2 cells expressing either CLK alone or CLK coexpressed with CK1α. We observed slower-migrating CLK isoforms on a regular SDS-PAGE gel (*SI Appendix*, Fig. S3), likely representing phosphorylated CLK. Phos-Tag SDS-PAGE gel (53) was used to enhance phosphorylation-dependent mobility shift (Fig. 3 *E* and *F*). In addition, to test whether CK1α catalytic activity is responsible for the observed mobility shift, we coexpressed CLK with either CK1α(WT) or CK1α(K49R), a kinase-dead variant (50). We observed substantial slower-migrating CLK species in the presence of CK1α(WT). The amount of slower-migrating species was significantly reduced with CK1α(K49R) coexpression. These results indicate that CK1α kinase activity is required for CLK mobility shift.

To specifically determine whether CLK(S13) is phosphorylated by CK1α, we leveraged mass spectrometry (MS) to identify CK1α-dependent phosphorylation sites of CLK expressed in *Drosophila* S2 cells. This cell culture system has previously been used to map physiologically relevant phosphorylation sites on *Drosophila* PER (47, 54, 55), TIM (56) and CLK (51, 57). We coexpressed CLK tagged with FLAG epitope with either CK1α(WT) or kinase dead CK1α(K49R) in S2 cells and performed FLAG affinity purifications prior to MS analysis. We identified eight phosphorylation sites on CLK [*SI Appendix*, Table S2 and (58)]. Among them, we identified four sites that exhibited elevated phosphopeptide abundance when coexpressed with CK1α(WT) as compared to CK1α(K49R) [Fig. 3*G* and (58)]. These CK1α-dependent sites include S13, which is next to the bHLH DNA binding domain (35, 36); S258 and S311 next to PAS B protein binding domain (35, 36); S476 next to the nuclear localization signal (NLS) (31).

Finally, to further validate the phosphorylation of S13, we generated a S13 phosphospecific antibody (α-pS13) and assayed CLK(S13) phosphorylation in protein extracts of *Drosophila* S2 cells expressing *Clk*-V5 (WT) with or without *ck1α* (Fig. 3 *H* and *I*). Immunoblotting showed that CLK(pS13) significantly increased when *ck1α* was coexpressed (Fig. 3*H*, lanes 1 and 2). Importantly, there was little to no α-pS13 signal detected in extracts of S2 cells coexpressing *Clk*(S13A) and *ck1α* (Fig. 3 *H*, lane 3, *Top*), suggesting that α-pS13 antibody is phosphospecific. Taken together, our results strongly support that CLK(S13) is a CK1α*-*dependent phosphorylation site.

### Flies Harboring Mutations at CLK(S13) Display Altered Circadian Behavioral and Molecular Output at 25 °C.

**Fig. 4.**
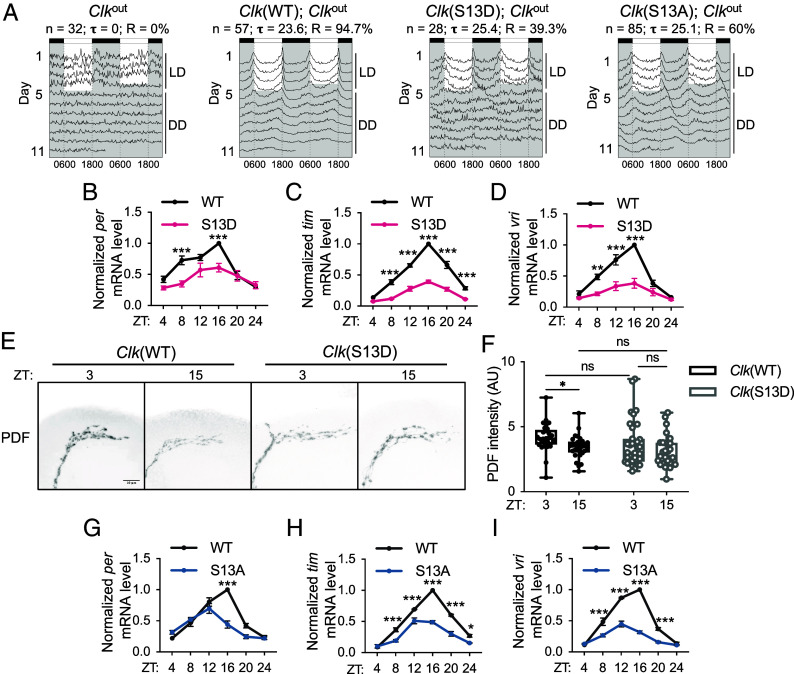
Flies expressing CLK(S13) variants display altered circadian behavioral and molecular output at 25 °C. (*A*) Double-plotted actograms of flies harboring two copies of transgenes expressing *Clk(WT)* or *Clk* with altered S13, a CK1α-dependent phosphorylation site, in *Clk*^out^ background. Average activity of each genotype was plotted using FaasX. n represents the sample size; Tau (τ) represents the average period length of the indicated group of flies in constant darkness (DD). R represents percentage of flies that are rhythmic. Flies were entrained for 4 d in 12 h:12 h LD and then *Left* in DD for 7 d. (*B*–*D*) Steady state daily mRNA expression of CLK targets (*per*, *tim*, and *vri*) in heads of *Clk*(WT); *Clk*^out^ and *Clk*(S13D); *Clk*^out^ flies. Flies were entrained in 12 h:12 h LD cycles at 25 °C and collected on LD3 at indicated time-points (ZT) (n = 3). Error bars indicate ± SEM. (*E*) Representative confocal images of dorsal projection of sLN_v_s neurons in adult fly brains stained with α-PDF (C7). Scale bar [merged image in *Clk*(WT) ZT3] represents 10 μm. Flies were entrained for 4 d in 12 h:12 h LD cycles and collected at the indicated times on LD4 for fixation and immunofluorescence analysis. (*F*) Box plot showing the quantification of PDF intensity in (*E*). Error bars indicate min to max. (*G*–*I*) Steady state mRNA expression of CLK targets (*per*, *tim*, *vri*) in heads of *Clk*(WT); *Clk*^out^ and *Clk*(S13A); *Clk*^out^ flies. Flies were entrained in 12 h:12 h LD cycles at 25 °C and collected on LD3 at indicated time-points (ZT) (n = 3). Error bars indicate ± SEM, ***P < 0.001, **P < 0.01, *P < 0.05, two-way ANOVA and Šídák’s post hoc test.

So far, our results suggest that S13 phosphorylation reduces CLK transcriptional activity. We also show that temperature-sensitive AS at cold temperature led to increased production of CLK-cold that lacks this inhibitory S13 phosphorylation, thus promoting CLK target mRNA expression in the cold. To characterize the function of CLK(S13) phosphorylation in vivo, we generated transgenic fly lines expressing nonphosphorylatable CLK(S13A) or phosphomimetic CLK(S13D) variants. These mutated *Clk* genes are expressed under endogenous *Clk* promotor (51). p{*Clk*(X)-V5} (X represents WT, S13A or S13D variants) transgenic fly lines were crossed into *Clk*^out^ background (51) to remove endogenous *Clk* expression. Next, we monitored daily locomotor activity rhythms of *Clk* transgenic flies, given it is a robust behavioral output of the circadian clock (59) (Fig. 4*A* and *SI Appendix*, Table S3). Flies were entrained for 4 d in 12 h:12 h light:dark (LD) cycles followed by release in 7 d in constant darkness (DD) at 25 °C to assess free-running rhythms. As expected, *Clk*^out^ null mutant exhibited arrhythmic locomotor activity in DD, similar to published results (51). *Clk*^out^ flies expressing two copies of *Clk*(WT) transgene displayed robust daily activity rhythms with a ~24-h period, indicating effective rescue of the arrhythmic *Clk*^out^ mutation (Fig. 4*A* and *SI Appendix*, Table S3). Even one copy of *Clk*(WT) transgene was sufficient for the rescue of arrhythmicity of the *Clk*^out^ null mutant, although two copies of *Clk*(WT) transgenes resulted in stronger behavioral rhythm. As compared to *Clk*(WT), *Clk*(S13D) flies with two copies of the transgene showed significantly dampened rhythm and period-lengthening of 1.2 h (*P* < 0.001, One-Way ANOVA), while *Clk*(S13D) flies with one copy of the transgene were mostly arrhythmic. *Clk*(S13A) flies with two or one copy of the transgene also displayed period-lengthening by 0.9 to 1.1 h and reduced rhythmicity as compared to *Clk*(WT) flies (two copies: *P* < 0.001; one copy: *P* < 0.001, One-Way ANOVA). Finally, both *Clk*(S13D) and *Clk*(S13A) can rescue arrhythmic *Clk*^out^ under LD, indicating light entrainment is not affected upon genetic manipulation of CLK(S13) phosphorylation. Taken together, our data suggest that CLK(S13) phosphorylation is required for robust circadian timekeeping.

To determine whether CLK(S13) phosphorylation-mediated downregulation in transcriptional activity observed in *Drosophila* S2 cells translates to whole animals, we quantified the mRNA of known CLK targets including *per*, *tim,* and *vri* in *Clk*(S13D) mutants at 25 °C. We observed significant reduction in levels and cycling amplitude of all mRNAs measured in *Clk*(S13D) mutants as compared to *Clk*(WT) flies, as determined by CircaCompare (60) (Fig. 4 *B*–*D* and *SI Appendix*, Table S4). The dampened mRNA oscillation in *Clk*(S13D) flies is consistent with dampened behavioral rhythmicity (Fig. 4*A*). It is important to highlight that the reduced expression of CLK targets in *Clk*(S13D) mutants is opposite of the higher CLK target gene expression when wild type flies were maintained at 10 °C rather than at 25 °C (compare Figs. 2*D* and 4 *B–D*). This supports that the S13 phosphorylation is a key regulatory event that fails to occur when flies express CLK-cold at 10 °C.

Since previous studies showed that phosphorylation-dependent reduction in CLK stability (49) is also a plausible mechanism to reduce CLK transcriptional activity, we wanted to rule out the possibility that CK1α can modify CLK stability (*SI Appendix*, Fig. S4 *A* and *B*). We measured CLK degradation independent of TTFLs by performing cycloheximide (CHX) chase assays in *Drosophila* S2 cells expressing CLK in the presence or absence of CK1α. We observed similar rates of CLK protein degradation in the two conditions, suggesting that CK1α does not regulate CLK stability. In agreement with S2 cell results, we did not observe significant difference in CLK abundance between *Clk*(WT) and *Clk*(S13D) flies maintained in LD cycles (*SI Appendix*, Fig. S4 *C* and *D*).

We next sought to explore potential clock output that mediates impaired behavioral rhythmicity in *Clk*(S13D) mutant. Pigment-dispersing factor (PDF), a key neuropeptide in clock neuronal circuits, has been shown as an important molecular clock output to regulate rhythmic locomotor activity in flies (61). PDF level is modulated by a number of CLK target genes, including *vri* (62), *hormone receptor-like 38* (HR38), (63) and *stripe* (63). Because we observed significant alteration of CLK target genes in *Clk*(S13D) flies (Fig. 4 *B*–*D*), we hypothesized that altered diurnal changes of PDF level at the dorsal projection of sLN_v_ neurons may cause dampened behavioral rhythms in *Clk*(S13D) mutant. We monitored PDF level using whole-mount immunocytochemistry, and observed that PDF exhibits diurnal changes in abundance in *Clk*(WT) flies, which is consistent with previous studies (62, 64) (Fig. 4 *E* and *F*). Diurnal changes in PDF abundance are abolished in *Clk*(S13D) mutant, despite normal behavioral rhythmicity in LD cycles. This is in agreement with previous findings indicating rhythmic PDF level is not required for maintenance of behavioral rhythmicity in LD (61, 62). Our results do not rule out that PDF rhythms are phase-shifted such that the difference observed in *Clk*(WT) flies at ZT3 and ZT15 was not observed in *Clk*(S13D) flies. This further supports the crucial role of S13 phosphorylation in maintaining diurnal changes of PDF level and therefore robust locomotor activity rhythms that do not occur when flies express CLK-cold at 10 °C.

In addition to *Clk*(S13D) flies, we also assayed CLK target gene expression in *Clk*(S13A) mutants. In contrary to what we expect based on our observation of elevated transcriptional activity of CLK(S13A) variant determined by *per-luc* reporter assay in *Drosophila* S2 cells (Fig. 3*A*), we observed that the expression of CLK target mRNAs in *Clk*(S13A) flies was also reduced when compared to *Clk*(WT) (Fig. 4 *G*–*I* and *SI Appendix*, Table S4). This discrepancy between the extent to which the *Clk*(S13A) mutation impacts CLK target gene expression in tissue culture vs. in whole animals is further explored in the following sections.

### CLK(S13) Phosphorylation Is Required for Robust Circadian Timekeeping at Cold Temperature.

**Fig. 5.**
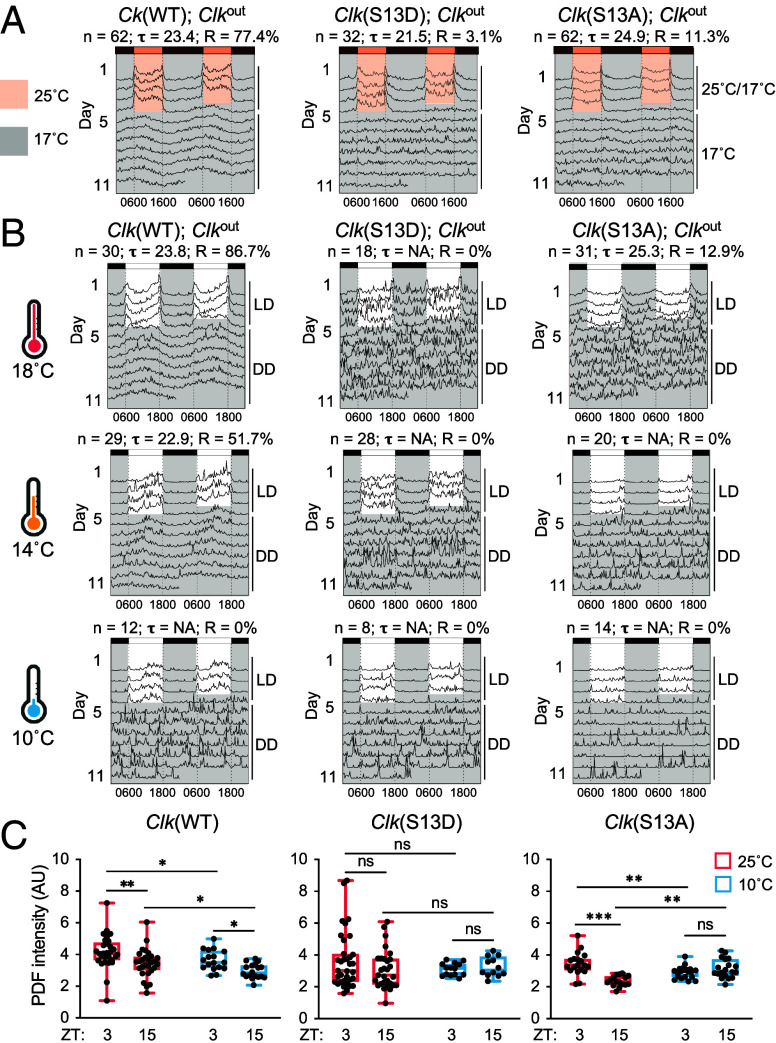
CLK(S13) phosphorylation is required to maintain behavioral rhythmicity at colder temperature. (*A* and *B*) Double-plotted actograms of flies harboring two copies of transgenes for *Clk*(WT)*, Clk*(S13A), or *Clk*(S13D). Average activity of each genotype was plotted using FaasX. n represents the sample size; Tau (τ) represents the average period length of the indicated group of flies in constant temperature. R represents percentage of flies that are rhythmic. For (*A*), flies were entrained for 4 d in 10 h:14 h 25 °C/17 °C (DD) and then released into constant 17 °C (DD) for 7 d. For (*B*), flies were entrained for 4 d in 12 h:12 h LD and then released into the indicated temperature for 7 d in DD. (*C*) Quantification of PDF intensity in dorsal projection of sLN_v_s neurons in adult fly brains stained with α-PDF (C7). Flies were entrained for 4 d in 12 h:12 h LD cycles and collected at the indicated times and temperature on LD4 for fixation and immunofluorescence analysis. Error bars indicate min to max, *P < 0.05, **P < 0.01, ***P < 0.001, two-way ANOVA.

Temperature can entrain circadian clocks (65, 66). Given the temperature-sensitive nature of *Clk* splicing (Fig. 1 *B* and *C*), we hypothesize AS of *Clk* can mediate temperature entrainment via regulating the availability of S13 residue for phospho-regulation. Therefore, *Clk*(S13A) and *Clk*(S13D) flies are expected to exhibit compromised temperature entrainment. To test this, we monitored daily locomotor activity rhythms of *Clk* transgenic flies for 4 d in 10 h:14 h 25 °C/17 °C temperature cycles in DD (67) followed by release in 7 d in 17 °C constant temperature to assess free-running rhythms (Fig. 5*A* and *SI Appendix*, Fig. S5*A*). We observed anticipation of cold phase in *Clk*(S13D) and *Clk*(S13A) flies (*SI Appendix*, Fig. S5*A*), which is a clear indication of thermic entrainment. Surprisingly, free-running rhythms are compromised in *Clk*(S13D) and *Clk*(S13A) after 4 d of thermic entrainment. To rule out the possibility that reduced rhythmicity at 17 °C is due to incomplete entrainment, we employed photic entrainment using LD cycles under several temperatures lower than 25 °C and monitored locomotor activities in DD (Figs. 4*A* and 5*B* and *SI Appendix*, Fig. S5*B*). *Clk*(WT) flies were rhythmic under 18 °C with similar period length as compared to 25 °C due to temperature compensation, while *Clk*(S13D) and *Clk*(S13A) both displayed reduced rhythmicity similar to that entrained by temperature steps (Fig. 5*A*). Under 14 °C, over 50% *Clk*(WT) individuals remained rhythmic, as seen in previous findings (68). However, *Clk*(S13D) and *Clk*(S13A) became completely arrhythmic. Moreover, we observed gradual phase-advanced evening activity peak for *Clk*(WT) under LD as we lowered temperature from 18 to 10 °C, a behavioral response for seasonal adaptation (13, 69). *Clk*(S13D) and *Clk*(S13A) did not show an obvious evening peak under LD at 10 to 18 °C. Together, these data suggest dynamic phosphorylation of CLK(S13) is required for the adaptation of behavioral rhythms to cold temperature, but not for temperature entrainment.

Given reduced behavioral rhythmicity of *Clk*(S13) mutants at cold temperature, we hypothesize that diurnal changes of PDF level, which is important for robust behavioral rhythm, are abolished in *Clk*(S13) mutants. We observed lower PDF level at the dorsal projection of sLN_v_ neurons at 10 °C as compared to 25 °C as well as diurnal changes (ZT3 vs. ZT15) of PDF level in *Clk*(WT) flies even under 10 °C (Fig. 5*C*), consistent with previous findings (69). However, changes in diurnal PDF level at 10 °C were abolished in *Clk*(S13D) flies, with no significant reduction at 10 °C as compared to 25 °C. In *Clk*(S13A) flies, diurnal changes in PDF level at 10 °C were also abolished, with a significant decrease at ZT3 (25 °C vs. 10 °C) but an increase at ZT15 (25 °C vs. 10 °C), suggesting compromised response of PDF level to cold temperature. Our results align with the model that S13 phospho-regulation mediates a large part of the effect of temperature-sensitive AS of *Clk*. Since S13 phosphorylation only occurs in CLK-long, *Clk*(S13D) flies are not expected to exhibit reduced PDF levels when the temperature is lowered (Fig. 5*C*). Conversely, *Clk*(S13A) flies resembling flies expressing CLK-cold should exhibit lower levels of PDF as compared to *Clk*(WT) independent of the temperature (*SI Appendix*, Fig. S6). In addition, we noticed *Clk*(S13A) flies displayed diurnal changes in PDF level at 25 °C (Fig. 5*C*), which helps to explain better rhythmicity of *Clk*(S13A) flies at 25 °C as compared with *Clk*(S13D) flies (Fig. 4*A*). Taken together, our data support temperature-dependent availability of S13 residue for phospho-regulation can act through PDF to regulate behavioral rhythms at cold temperature.

### CLK(S13) Phosphorylation Reduces CLK Occupancy at CLK Target Gene Promoters.

**Fig. 6.**
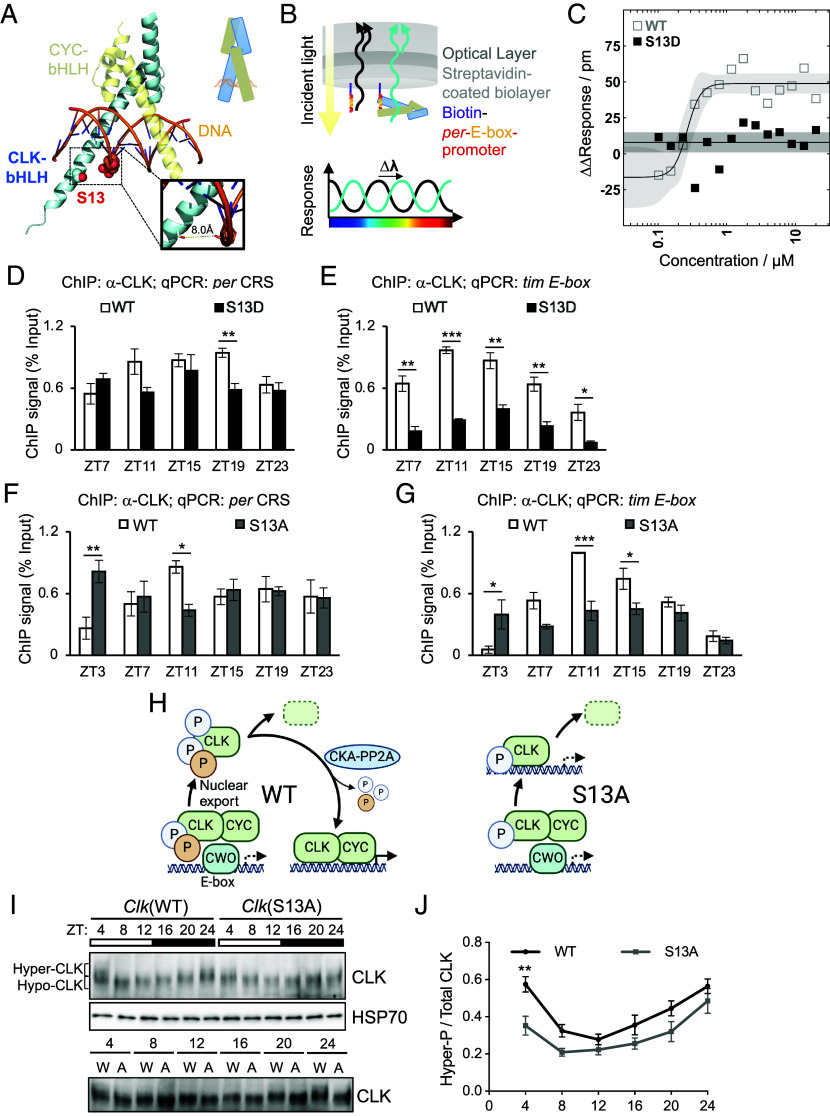
CLK(S13) phosphorylation modulates CLK-DNA binding. (*A*) Model of *Drosophila* CLK (cyan)-CYC (yellow) bHLH heterodimer in complex with DNA (orange), with the side chain of S13 and the phosphate backbone of a proximal *E-box* nucleotide shown as spheres. The S13 hydroxyl oxygen atom, along with the oxygen atoms in the phosphate backbone, are shown in red. The *Inset* shows a close-up view and the estimated distance between the two oxygen atoms. The model was generated by superimposing an AlphaFold-predicted structure of *Drosophila* CLK-bHLH (aa 1-71) with the crystal structure of human CLK and BMAL1 in complex with *E-box* DNA (PDB 4H10). A cartoon representation is shown in the *Upper Right*. (*B*) Design of the Biolayer Interferometry (4) experiment. Biotin-labeled *per* promoter DNA was immobilized onto streptavidin-coated biosensors. CLK-bHLH-DNA interactions led to an increase in the effective thickness in the biolayer and a change in interference wavelength. (*C*) Quasi-steady state signal response of CLK-bHLH-DNA binding in the presence (black, filled) and absence (gray, hollow) of the phosphomimetic S13D mutation. Solid lines and shaded areas show fits and 95% prediction interval to the 4-parameter Hill equation for CLK-bHLH-WT and nonbinding baseline for CLK-bHLH-S13D. (*D*–*G*) ChIP assays using fly head extracts comparing CLK occupancy at *per* and *tim* promoter of indicated fly genotypes on LD3 at indicated time-points (ZT) after entrainment in 12 h:12 h LD at 25 °C. CLK-ChIP signals were normalized to % input. ChIP signals for two intergenic regions were used for nonspecific background deduction (*D* and *E*, n = 3; *F* and *G*, n = 4). Error bars indicate ± SEM, ***P < 0.005, **P < 0.01, *P < 0.05, two-way ANOVA and Šídák’s post hoc test. (*H*) Model describing the alteration of the molecular clock in flies expressing CLK(S13A) variant. In WT flies, upon CLK removal from DNA promoted by PER-dependent phosphorylation and CWO-dependent mechanisms, S13 phosphorylation prevents CLK from binding back to DNA. CLK then undergoes nuclear export and further phosphorylation (31). Hyperphosphorylated CLK is either targeted for degradation or dephosphorylated by CKA-PP2A complex to replenish the pool of hypophosphorylated CLK (30, 31). Dephosphorylated CLK and newly translated, hypophosphorylated CLK then promotes transcription of clock-controlled genes. In S13A flies, after initial CLK removal from DNA by CWO, increased CLK(S13A)-DNA binding affinity leads to premature binding of transcriptionally inactive CLK and DNA. This leads to a decrease in CLK(S13A) nuclear export, hence reducing the replenishment of transcriptionally active CLK in the next cycle. PER and TIM are not depicted for simplicity. Created with BioRender.com licensed to the lab of J.C. Chiu. (*I*) Western blots comparing CLK protein profiles in heads of *Clk*(WT); *Clk*^out^ and *Clk*(S13A); *Clk*^out^. Flies were entrained for 4 d in 12 h:12 h LD and collected at the indicated times on LD3. Brackets indicate hypo- and hyperphosphorylated CLK isoforms. α-HSP70 was used to indicate equal loading and for normalization. *Bottom* blot also detects CLK in the same two genotypes (W for WT and A for S13A) but the samples for each timepoint (ZT) were ran side by side to facilitate comparison of mobility shift. (*J*) Quantification of hyperphosphorylated/total CLK. The top half of the CLK signal shown at ZT4 in *Clk*(WT) flies (lane 1) is used as a reference to classify CLK isoforms as hyperphosphorylated (n = 4). Error bars indicate ± SEM, **P < 0.01, two-way ANOVA and Šídák’s post hoc test.

Now that we showed that CLK(S13) phosphorylation is important for maintenance of circadian rhythms in a physiological range of temperature, we further investigated the molecular basis of CLK(S13) phosphorylation in regulating circadian rhythms by characterizing the impact of S13 phosphorylation in modulating CLK-DNA interactions in vitro and in flies. We overlaid an AlphaFold (70) model of *Drosophila* CLK-bHLH (aa1-71 of CLK-long including the bHLH domain) to the crystal structure of human CLOCK-BMAL1-DNA (71) and found plausible contacts between S13 and the negatively charged DNA backbone (Fig. 6*A*). This hints at phosphorylation being a direct modulator of CLK-DNA binding by suppressing CLK-DNA interactions via charge–charge repulsion. To test this hypothesis, we expressed and purified aa1-71 fragment of CLK-bHLH with and without the S13D mutation from *Escherichia coli* (*SI Appendix*, Fig. S7 *A* and *B*) and measured their binding affinity to a 21-bp *per* promoter as bait (72) using biolayer interferometry (BLI) (73) (Fig. 6*B*). The EC_50_ between WT CLK-bHLH and DNA binding was estimated to be 0.27 μM (95% CI = [0.19, 0.37], Fig. 6*C*). Importantly, introduction of the phosphomimetic S13D mutation abolishes the binding of CLK-bHLH to DNA (Fig. 6*C*), demonstrating the potent inhibition of S13 phosphorylation on the ability of CLK to bind to DNA and act as a transcription factor. To demonstrate the specificity of the CLK-*per* promoter interaction, we performed a control experiment where the DNA is composed of a scrambled DNA sequence. We observed a reduction in CLK binding affinity on scrambled DNA compared to the 21-bp *per* promoter sequence, suggesting that the CLK-DNA binding in our BLI assay is selective for CLK target gene promotors (*SI Appendix*, Fig. S7*C*).

We next performed CLK-ChIP followed by qPCR using extracts from adult fly heads to further determine CLK-DNA binding in fly tissues collected at 25 °C (Fig. 6 *D* and *E*). We observed significantly lower CLK occupancy in *Clk*(S13D) mutants as compared to *Clk*(WT) flies at ZT19 at the *per CRS* (Fig. 6*D*). CLK occupancy at the *tim E-box* is also significantly lower in *Clk*(S13D) mutants at all time-points tested (Fig. 6*E*). Together with data from in vitro experiments (Fig. 6 *A*–*C*), our results revealed that CLK occupancy at CLK target gene promoters decreases upon CLK(S13) phosphorylation, which explains lower expression of CLK target genes in *Clk*(S13D) flies.

**Fig. 7.**
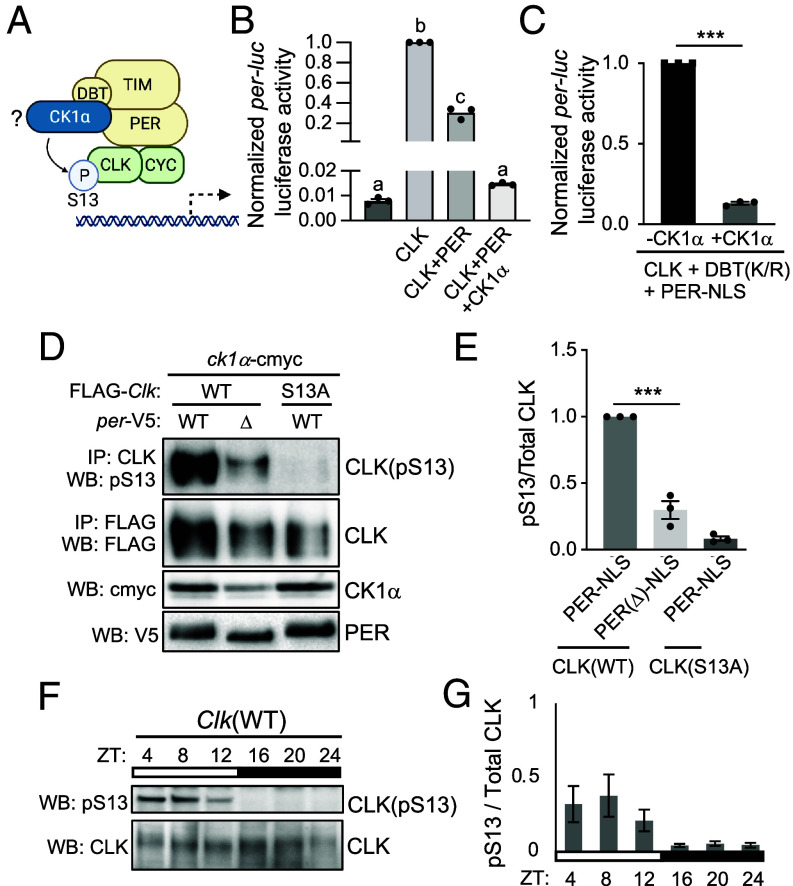
PER-DBT scaffolding promotes CK1α-dependent CLK(S13) phosphorylation. (*A*) Schematic illustrating the PER-DBT scaffolding model first proposed by Yu et al. (28). Created with BioRender.com licensed to the lab of J.C. Chiu. (*B* and *C*) *per-E-box-luciferase* (*per-luc*) reporter assay performed in S2 cells. (*B*) The fold activation of *per-luc* were graphed. Error bars indicate ± SEM (n = 3). One-way ANOVA and Tukey’s post hoc test. (*C*) Luciferase activities were normalized to CLK+DBT(K/R)+PER-NLS. Error bars indicate ± SEM (n = 3). (*D*) *Drosophila* S2 cells were transfected with indicated plasmids. Protein extracts were incubated with α-FLAG resin. Total CLK isoforms, pCLK(S13), PER, and CK1α protein levels were analyzed by Western Blotting with indicated antibodies. (*E*) Bar graph showing relative CLK pS13 levels in (*D*) normalized to total CLK level. Error bars indicate ± SEM (n = 3), ***P < 0.001, one-way ANOVA and Šídák’s post hoc test. (*F* and *G*) Fly heads of the specified genotypes were collected at the indicated times on LD3 after 2 d of entrainment. Extracted proteins (lysate) were subjected to western blotting with α-CLK(pS13) directly without IP. Total CLK proteins are shown in the *Bottom* panel and used for normalization and quantification shown in (*G*) (n = 3).

In the case of *Clk*(S13A) mutant, CLK-ChIP qPCR showed significantly higher CLK occupancy at the *per CRS* and *tim E-box* at ZT3 (Fig. 6 *F* and *G*). Increased CLK-DNA binding in early day in *Clk*(S13A) mutant (ZT3, Fig. 6 *F* and *G*) resembles our observation of higher CLK-DNA binding in WT flies at 10 °C (ZT4, Fig. 2 *A* and *B*). These results support our model that AS allows CLK-cold to escape inhibitory phosphorylation at S13 to promote CLK-DNA binding. Surprisingly, CLK occupancy at ZT11 (*per CRS* and *tim E-box*) and ZT15 (*tim E-box*) in *Clk*(S13A) mutant is significantly lower as compared to that in *Clk*(WT). How might increased CLK-DNA binding in early morning contribute to a reduction of CLK-DNA binding in the beginning of the night? Upon CLK removal from DNA by CWO (74) and nuclear export (31), a fraction of CLK undergoes further phosphorylation followed by degradation (31) (Fig. 6*H*). Whereas another fraction of CLK is dephosphorylated by CKA-PP2A complex to replenish the pool of hypophosphorylated CLK for the next round of CLK-activated transcription (30). We hypothesize in *Clk*(S13A) mutants, CLK is still removed off DNA by CWO in late night. Without S13 phosphorylation, CLK would therefore exhibit premature DNA binding activity in early morning. These transcriptionally inactive CLK proteins cannot be exported out of the nucleus to be dephosphorylated at other residues and are eventually degraded in the nucleus. Without replenishing transcriptionally active CLK via nuclear export and dephosphorylation, CLK occupancy at circadian promoters in the beginning of the night is reduced (Fig. 6 *F* and *G*; ~ZT11-ZT15). To test this hypothesis, we analyzed daily CLK phosphorylation in *Clk*(S13A) (Fig. 6 *I* and *J*). As we expected, CLK phosphorylation level in *Clk*(S13A) was significantly reduced in early day (ZT3) as compared to *Clk*(WT). This is likely due to increased DNA-binding activity and the inability of CLK to be exported to the cytoplasm for further phosphorylation prior to degradation. Notably, reduced CLK occupancy at circadian promoters when CLK target transcription peaks (ZT11-15) (Fig. 6 *F* and *G*) is consistent with lower CLK target mRNA levels in *Clk*(S13A) mutants (Fig. 4 *G*–*I*). We cannot however rule out the possibility that additional feedback mechanisms absent in tissue culture system could have contributed to the discrepancy between reduced CLK target gene expression in *Clk*(S13A) flies (Fig. 4 *G*–*I*) and increased transcriptional activity of CLK(S13A) in S2 cells (Fig. 3*A*). Nonetheless, the timing of S13 phosphorylation primarily in the morning (Fig. 7 *F* and *G*), as shown by detection of S13 phosphorylation in flies using phosphospecific antibody also supports our model.

### PER-DBT Interaction Influences CK1α-Dependent Downregulation of CLK Transcriptional Activity.

Finally, we sought to determine the molecular requirements for CK1α to phosphorylate CLK(S13). It has been proposed that PER-TIM repressor complexes recruit yet uncharacterized kinases for timely CLK phosphorylation to enhance repression (32, 75). Since CK1α has been shown to interact with PER in both the cytoplasm and the nucleus (50), we hypothesize that PER promotes CK1α-dependent phosphorylation of CLK (Fig. 7*A*). We first performed *per-luc* reporter assay to measure CLK transcriptional activity in S2 cells expressing CLK and PER in the absence or presence of CK1α (Fig. 7*B*). As expected, PER expression down-regulates CLK transcriptional activity. CK1α coexpression further reduced CLK transcriptional activity, indicating an enhanced PER repression via PER-dependent CK1α phosphorylation of CLK. We observed no significant difference between baseline luciferase activity and cells expressing CLK and PER in conjunction with CK1α, suggesting that PER and CK1α together essentially abolished transcriptional activity of CLK.

CK1α regulates PER repression activity by favoring PER nuclear entry and promoting PER-DBT interaction and phosphorylation-dependent degradation (50). To remove the confounding effect of CK1α on PER nuclear localization and degradation in our luciferase assay, we therefore expressed nuclear localization sequence (NLS)-tagged PER (PER-NLS) and DBT(K/R), a kinase-dead DBT variant (76) to specifically examine the role of CK1α in modulating CLK activity (Fig. 7*C*). We observed significant reduction in CLK-dependent *per-luc* activity upon CK1α coexpression with PER-NLS and DBT(K/R). This suggests that in addition to the regulation of CK1α on PER nuclear entry and stability, the enhanced PER repression of CLK seen in Fig. 7*B* is also mediated through CLK phosphorylation by CK1α.

To directly determine whether PER-DBT scaffold promotes CLK(S13) phosphorylation by CK1α, we assayed CLK(S13) phosphorylation in protein extracts of *Drosophila* S2 coexpressing FLAG-*Clk* and *ck1α* in combination with either *per*(WT) or *per*(Δ), a *per* mutant encoding a variant lacking PER-DBT binding domain (77) (Fig. 7 *D* and *E*). Western blotting with S13 phosphospecific antibody showed that CLK(pS13) was significantly reduced when *per*(Δ) is coexpressed, as compared to *per*(WT) (Fig. 7*D*, lane 1-2). As expected, little to no CLK(pS13) signal was detected in *Clk*(S13A) (Fig. 7*D*, lane 3), showing the specificity of α-pS13.

In flies, PER interacts with CLK upon nuclear entry (33). PER-DBT can promote CLK(S13) phosphorylation for CLK removal from DNA in the late night. Alternatively, CLK(S13) phosphorylation can occur in the early morning when CLK is removed from DNA, which prevents premature DNA binding of CLK. To determine the timing of CLK(S13) phosphorylation, we detected CLK(S13) phosphorylation level by Western blots using α-pS13 in head extracts from flies collected over a daily cycle (Fig. 7 *F* and *G*). We observed that S13 phosphorylation occurs in the daytime (ZT4, ZT8, and ZT12), supporting the function of CLK(S13) phosphorylation as a prevention of premature CLK binding to DNA. Together, our results support that PER-DBT serves as a scaffold to enhance CK1α-dependent CLK(S13) phosphorylation to sustain off-DNA state of CLK.

## Discussion

**Fig. 8.**
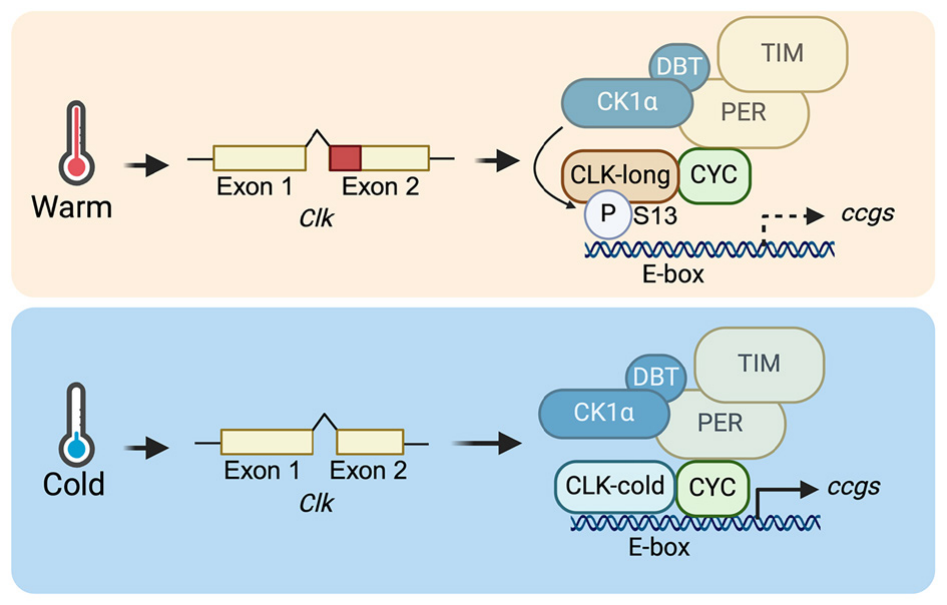
Model illustrating the regulation of the molecular clock by temperature-sensitive alternative splicing of *Clk*. *Top* panel: At warm temperature (25 °C), CLK-long isoform harboring S13 residue is expressed, due to alternative 3′ splice site selection of exon 2 of *Clk*. CLK-cold is also expressed but not shown to simplify the model. PER-DBT scaffolding promotes CK1α-dependent phosphorylation of CLK(S13) and reduces CLK-DNA binding. *Bottom* panel: At cold temperature (10 °C), expression of CLK-cold isoform lacking S13 residue increases, therefore lowering the impact of S13 inhibitory phosphorylation. As a result, this leads to elevated mRNA expression of CLK targets under low temperature. Created with BioRender.com licensed to the lab of J.C.C.

In this study, we report temperature-sensitive AS of the core clock gene, *Clk*. Under cold condition, CLK-cold expression is elevated and it displays higher transcriptional activity as compared to the canonical CLK-long isoform. S13 is a phosphorylation site among the four amino acids deleted in CLK-cold protein. Combining the results from a series of molecular and behavioral analyses of transgenic flies expressing nonphosphorylatable *Clk*(S13A) and phosphomimetic *Clk*(S13D) mutants, we formulated a model describing AS of *Clk* transcripts in regulating the response of the molecular clock to temperature changes (Fig. 8). At higher temperature (25 °C), CLK-long harboring S13 residue is expressed. CK1α relies on PER-DBT as a scaffold to phosphorylate CLK(S13), which prevents CLK-DNA binding in the early morning. At low temperature (10 °C), the ratio of *Clk-*long*/Clk-*cold expression is altered with *Clk-*cold expression becoming significantly higher compared to its expression level at warmer temperatures. Since CLK-cold lacks S13, it enables the molecular clock to escape regulation by CK1α-dependent S13 phosphorylation at cold temperature, essentially lowering the impact of S13 phospho-regulation. As a result, mRNA expression of CLK target genes is enhanced at low temperature.

We observed daily rhythms of *Clk* transcripts under environmental temperature cycles in ectothermic flies (Fig. 1). Mice and humans, despite being endothermic, display body temperature rhythm of a few degrees that is shown to entrain peripheral clocks (78, 79). Temperature-sensitive AS has been identified in over 1,000 exons (12), including genes involved in general transcription (80) and regulating stability of core clock protein mPER1 (81). Future studies are required to fully understand the role of temperature-sensitive AS on the entrainment of peripheral clocks by body temperature rhythms. It is also interesting to point out that cold temperature also induces mRNA expression of several CLOCK target genes in human cardiomyocytes (82). Similarly, lowered body temperature during hibernation of brown bears also results in elevated mRNA level of a CLOCK target gene, *cry2* (83). Although AS of mammalian *Clock* has been previously identified (84), whether AS of mammalian *Clock* mediates mRNA expression of its targets in a temperature-sensitive manner remains to be investigated.

We show that increased transcriptional activity of CLK-cold promotes CLK target mRNA expression at cold temperature (Fig. 2). Previous studies suggest that cold-induced intron splicing of *per* and accumulation of *tim*-SC transcripts promote organismal adaptation to cold temperature (13, 23, 25). It is possible that the elevated transcriptional activity of CLK due to AS of *Clk* transcripts can promote cold adaptation by increasing *per* and *tim*-SC mRNA level. In addition, CLK-cold could regulate cold adaptation through PDF, which can down-regulate EYES ABSENT, a seasonal sensor protein that integrates temperature and photoperiodic signals (69, 85). As our lab previously showed that PDF level decreases in flies at 10 °C, which we confirmed in this study, one potential mechanism by which CLK-cold regulates PDF is to increase the expression of HR38 and/or SR, two CLK targets that can inhibit PDF expression (63). Moreover, Li et al. (86) recently identified that DN1a dorsal neurons can modulate locomotor activity and sleep distribution in response to temperature changes. Since both *Clk*-long and *Clk*-cold are expressed in DN1 neurons (38), it is possible that AS of *Clk* transcripts may contribute to the temperature sensing function of DN1a neurons. In addition, since central clock neurons in flies rely on peripheral thermoreceptors to exhibit temperature responses (87, 88), AS of *Clk* in central clock neurons, e.g., DN1a neurons, may be a downstream responder of peripheral thermoreceptor signaling. Another possibility is that temperature-sensitive AS of *Clk* is more critical for peripheral clocks than central clocks for in response to temperature. Brief inspection of genomic *Clk* of several other *Drosophila* species indicates that they also have potential alternative 3’ splice sites that can produce both *Clk*-long and *Clk*-cold. This indicates the adaptative value of *Clk* AS is not limited to *D. melanogaster*, but also in other *Drosophila* species, such as cold-adapted *Drosophila montana* and an agricultural pest, *Drosophila suzukii*. To further understand how flies adapt to cold through AS of *Clk*, future studies are necessary to uncover how daily rhythmic transcriptome is altered in the cold.

Within the four amino acids that are spliced out in CLK-cold, we identified S13 as a phosphorylation site adjacent to the bHLH domain that regulates CLK-DNA binding (Fig. 6). We hypothesize that flies expressing the nonphosphorylatable *Clk*(S13A) mutant would partially mimic the phenotype of flies under cold conditions. Indeed, CLK binding to DNA is elevated at early morning time in both *Clk*(S13A) mutant flies and flies at 10 °C (Figs. 2 *A* and *B* and 6 *F* and *G*). However, we also observed some discrepancies regarding CLK-DNA binding at later time-points in late day and early evening (Figs. 2 *A* and *B* and 6 *F* and *G*) as well as the mRNA levels of CLK targets (Figs. 2*D* and 4 *G–I*) between these two types of flies. It is important to note that *Clk* AS is certainly not the only mechanism mediating temperature responses of the molecular clock. For instance, under cold conditions, *per* and *tim* also undergo AS and alter their repressor activity on CLK, and these mechanisms are not at work in *Clk*(S13A) flies under warm temperature. Although we did not generate and analyze transgenic flies expressing only CLK-cold variant, we expect that such flies will exhibit elevated DNA binding activity of CLK and dampened behavioral rhythmicity similar to *Clk*(S13A) mutant at lower temperature. This expectation is based on our tissue culture data (Fig. 2*C*) and the importance of CLK(S13) phospho-regulation in maintenance of behavioral rhythmicity under different temperatures (Figs. 4*A* and 5). Future analysis of flies expressing specific *Clk* isoforms can provide further support to our model.

In addition to interpreting the molecular phenotype of flies at 10 °C, the characterization of *Clk*(S13A) mutant provided us with insights into the mechanism by which CLK transcriptional activity is repressed by phosphorylation. Our CLK-ChIP data showed no significant increase of CLK-DNA binding in *Clk*(S13A) mutant at late night (ZT23) as compared to *Clk*(WT) (Fig. 6 *F* and *G*), indicating CLK-DNA dissociation is not affected when CLK(S13) phosphorylation is abolished. Rather, CLK-DNA binding is increased in early morning (ZT3) in *Clk*(S13A) mutant, consistent with the timing of CLK(S13) phosphorylation in fly tissues (Fig. 7 *F* and *G*). We reasoned that upon S13 phosphorylation, CWO outcompetes CLK in *E-box* binding activity, preventing off-DNA CLK from binding back. This is supported by previous findings that PER, likely PER-dependent phosphorylation of CLK, is necessary for CWO to compete with CLK for *E-box* binding (74). It is somewhat surprising that elevated CLK-DNA binding at ZT3 in *Clk*(S13A) mutants does not enhance CLK target gene expression (Fig. 4 *G*–*I*). This supports the importance of other phosphorylation sites and/or other posttranslational modifications, such as CK2-dependent phosphorylation (49) and USP8-dependent deubiquitylation (89), in repressing transcriptional activity of CLK. In conclusion, the primary function of S13 phosphorylation is likely to prevent off-DNA CLK from prematurely associating with DNA in early morning, rather than dissociating CLK from DNA in the evening.

We provide evidence supporting the long-standing hypothesis proposed in 2006 that PER acts as a scaffold to deliver unknown kinases to directly repress transcriptional activity of CLK (28). DBT was first implicated as the CLK kinase (28, 77), while follow-up studies suggest kinase activity of DBT is not required for CLK phosphorylation (32). Rather, DBT together with PER act as the molecular scaffold for the phosphorylation-dependent repression on CLK. Our lab previously showed that CK1α interacts with PER in both the cytoplasm and the nucleus (50). Here, we showed that CK1α is a CLK kinase that requires the presence of PER-DBT complex to phosphorylate CLK at S13 (Figs. 3 and 7). As discussed above, CLK S13 phosphorylation inhibits CLK-DNA binding activity. In addition, casein kinase 1 (CK1)-dependent phosphorylation of repressor proteins has been proposed as conserved timing mechanisms in eukaryotic circadian clocks, despite different activator and repressor proteins employed (90). Together with findings in fungi (91–93) and mammals (94, 95), our data further suggest the CK1-scaffolding role of repressor proteins as additional conserved features in regulating eukaryotic circadian clocks.

In summary, we uncovered an interplay between temperature-sensitive AS and phosphorylation in modulating the activity of a master clock transcriptional activator. Many studies have been devoted to investigate the function of AS, as it is prevalent in 42 to 95% intron-containing genes across species (96–99). However, adjacent AS sites (<=18 bps) are usually overlooked due to their perceived minor influence in protein coding, despite the prevalence of adjacent AS sites in transcriptomes among several species (97, 100–102). Our current study provides an example of the importance of adjacent AS sites. Moreover, interplay between AS and phosphorylation has been shown to occur at different levels, including phosphorylation of splicing-related proteins (11, 12), AS of kinases and phosphatases (38, 103, 104) and inclusion of cassette exons with phosphorylation sites (105). Here, we provide an additional mechanism in which AS regulates protein function by removing amino acid(s) as substrate for phosphorylation encoded between adjacent AS sites.

## Materials and Methods

Detailed *Materials and Methods* are provided in *SI Appendix*, *SI Materials and Methods*.

### Fly Stocks.

*Drosophila* construct design and transformation of *Clk* transgene was performed as described by Mahesh et al. (51). Details are provided in *SI Appendix*.

### Luciferase Reporter Assay.

Luciferase reporter assay was performed as described by Darlington et al. (36). Details are provided in *SI Appendix*.

### ChIP and Quantitative PCR.

ChIP and quantitative PCR were performed as described by Kwok et al. (75). Details are provided in *SI Appendix*.

### CoIP, Western Blotting, and Antibodies.

Methods for coIP and western blotting and information about antibodies were as described by Lam et al. (50). Additional details are provided in *SI Appendix*.

### Phos-Tag SDS-PAGE.

Phos-Tag SDS-PAGE was performed as described by Lam et al. (50). Details are provided in *SI Appendix*.

### Identification of CLK Phosphorylation Sites by Mass Spectrometry.

Expression of CLK in *Drosophila* S2 cells, purification of CLK proteins, and subsequent mass spectrometry analysis were as described by Chiu et al. (54). Additional details are included in *SI Appendix*.

### Generating CLK(S13) Phosphospecific Antibodies.

CLK(S13) phosphospecific antibodies was generated as described by Chiu et al. (54). Details are included in *SI Appendix*.

### *Drosophila* Locomotor Activity Assay.

Fly activity monitoring was performed as described by Hidalgo et al. (69). Details are included in *SI Appendix*.

### Immunofluorescence.

Immunofluorescence was performed as described by Cai et al. (75). Details are provided in *SI Appendix*.

### Cycloheximide Chase Assay.

Cycloheximide chase assay was performed in *Drosophila* S2 cells as described by Lam et al. (50). Details are provided in *SI Appendix*.

### Biolayer Interferometry.

Biolayer interferometry to assay CLK-DNA binding was performed as described by Abdiche et al. (73). Additional details are included in *SI Appendix*.

### Statistical Analysis.

Statistical analyses were performed as described by Cai et al. (75). Details are included in *SI Appendix*.

## Supplementary Material

Appendix 01 (PDF)

## Data Availability

Raw data have been deposited in GitHub (https://github.com/ClockLabX/CLK_AS) (106). All other data are included in the manuscript and/or *SI Appendix*.
