# Supplementary material for: Alternative splicing of Clock transcript mediates the response of circadian clocks to temperature changes
Source: Proc Natl Acad Sci U S A. 2024 Dec 4;121(50):e2410680121. doi: 10.1073/pnas.2410680121 (PMC11648895; doi:10.1073/pnas.2410680121)
Supplement: Supplementary file 1 — Appendix 01 (PDF) [file pnas.2410680121.sapp.pdf]

PNAS

Supporting Information for
Alternative splicing of *Clock* transcript mediates the response of circadian clocks to temperature changes

Yao D. Cai[1†], Xianhui Liu[2†], Gary K. Chow[1], Sergio Hidalgo[1], Kiya C. Jackson[1], Cameron D. Vasquez[1], Zita Y. Gao[1], Vu H. Lam[1], Christine A. Tabuloc[1], Haiyan Zheng[3], Caifeng Zhao[3], Joanna C. Chiu[1]

[1]Department of Entomology and Nematology, College of Agricultural and Environmental Sciences, University of California Davis, One Shields Ave, Davis, CA 95616, USA.
[2]Cambridge-Suda Genomic Resource Center, Suzhou Medical College, Soochow University, Suzhou, China
[3]Biological Mass Spectrometry Facility, Robert Wood Johnson Medical School and Rutgers, the State University of New Jersey, Piscataway, NJ 08854, USA.
[†]These authors have contributed equally to this work.

Joanna C. Chiu
Email: jcchiu@ucdavis.edu

This PDF file includes:

Supporting text
Figures S1 to S7
Tables S1 to S5
SI References

Supporting text

SI Materials and Methods

Material availability

All unique/stable reagents generated in this study are available from the lead contact without restriction.

Data and code availability

The codes used in the data analysis and the datasets generated during the current study can be found at Github [https://github.com/ClockLabX/CLK_AS].

Experimental Models and Subject Details

Drosophila construct design and transformation

attB-P[acman]-*Clk* (15.5kb of the genomic sequence beginning ~8kb upstream and ending ~2.5kb downstream of *Clk* coding region) was kindly provided by Paul Hardin[1]. To introduce a V5 epitope tag in the C-terminus of the *Clk* coding region, a 4kb NheI-NotI *Clk* fragment was subcloned into pSP72 construct and sequences encoding V5 were introduced in frame by site-directed mutagenesis using Pfu Turbo Cx DNA polymerase (Agilent Technologies, Santa Clara, CA) (See Table S5 for mutagenic primer sequences). The resulting NheI-NotI *Clk*-V5 fragment was then used to replace the NheI-NotI *Clk* fragment in *attB*-P[acman]-*Clk* to generate *attB*-P[acman]-*Clk*-V5. PhiC31-mediated transgenesis[2] was used to generate w^{1118}; *Clk*(WT)-V5. Plasmids were injected into PBac{y[+]-attP-9A}VK00018 fly embryo (Bloomington #9736) (BestGene, Chino Hills, CA)[3]. Transformants were crossed with w^{1118}; +; Clk^{out} flies (Bloomington #56754)[1] to remove endogenous copies of *Clk* prior to behavioral and molecular analyses.

To generate flies expressing non-phosphorylatable (Serine (S) to Alanine(A)) or phosphomimetic (S to Aspartic acid (D)) *Clk* mutants, a 7kb NheI-SphI *Clk* fragment was subcloned into pSP72 plasmid where a NheI site were introduced to multicloning sites. After site-directed mutagenesis and confirmation by Sanger sequencing (GENEWIZ Inc, South Plainfield, NJ), the mutant variants of 7kb *Clk* fragments were used to replace the corresponding WT fragment in *attB*-P[acman]-*Clk*(WT)-V5. Transgenic flies were generated by Bestgene (Chino Hills, CA) as described above. Transgenic flies with one or two copies of *Clk*(WT), *Clk*(S13A), and *Clk*(S13D) transgenes were used in behavioral assays shown in Table S3 while

only flies with two copies of transgenes were used in all other experiments.

Method Details

RNA Extraction, cDNA Synthesis, RT-PCR, and quantitative PCR

RNA was extracted from approximately 30-50μl of fly heads using 3X volume TRI Reagent (Sigma-Aldrich, St. Louis, MO). 1/5 volume of 100% chloroform (Sigma-Aldrich) was added and incubated at room temperature for 10 minutes. Upper aqueous layer was recovered after spinning down at 13,000 rpm for 15 minutes. Same volume of 100% isopropanol was added and incubated at -20˚C overnight to precipitate RNA. After spinning down, RNA pellet was washed with 200μl 70% ethanol once, resuspended in 20μl 1X RQ1 buffer (Promega, Madison, WI), and treated with 2μl RQ1 DNase (Promega) at 37°C for 30 minutes prior to the incubation with 2μl RQ1 DNase stop solution (Promega) at 65˚C for 10 minutes. cDNA was generated from equal amount of RNA for each sample using Superscript IV (Thermo Fisher Scientific, Waltham, MA). Real-time PCR was performed using SsoAdvanced SYBR green supermix (Bio-Rad, Hercules, CA) in a CFX96 or CFX384 (Bio-Rad). Three technical replicates were performed for each of three biological qPCR replicates.

To confirm expression of *Clk* isoforms, RNA was extracted as above. cDNA was generated using gene-specific primer *Clk*(698R). PCR was then performed using *Clk*(1F) and *Clk*(101R) prior to resolving PCR products in 2% TBE gel. Bands were excised, and gel extracted for direct Sanger sequencing as well as TA cloning (Life technologies (Carlsbad, CA)) in combination with Sanger sequencing. Sequences for all primers are presented in Table S5.

Plasmids for *Drosophila* S2 cell culture

pAc-*Clk*(WT)-V5[4], *per-E-box-Luciferase*[5], *pCopia Renilla-Luciferase*[6], pAc-*per*(WT)-V5[7], pMT-*ck1α*(WT)-c-myc and pMT-*ck1α*(K49R)-FH[8] (FH denotes 3XFLAG-6XHis) were previously described. pAc-*Clk*-cold was generated by deleting 12bp encoding aa 13-16 of CLK-long from pAc-*Clk*(WT)-V5 using mutagenic primers in Table S5.

Drosophila S2 cell culture and transfection

Drosophila S2 cells and Schneider's *Drosophila* medium were obtained from Life Technologies. S2 cells were grown at 22˚C in Schneider's *Drosophila* medium supplemented with 10% Fetal Bovine Serum (FBS) (VWR, Radnor, PA) and 0.5% Penicillin/streptomycin (Sigma-Aldrich). For all cell culture experiments unless otherwise noted, S2 cells were seeded at 1 X 10^6 cells/ml in a 6-well plate and transfected using Effectene (Qiagen, Germantown, MD). For coimmunoprecipitation (coIP) assays in Fig. 3B, S2 cells were cotransfected with 0.8µg of pAc-*Clk*-V5-His and 0.8µg of pMT-*ck1α*-6Xc-myc, and induced with 500 µM $CuSO_4$ immediately after transfection. In control IPs to detect non-specific binding, cells were transfected with either pAc-*Clk*-V5-His or pMT-*ck1α*-6Xc-myc, in combination with pMT-FH empty plasmid to balance amount of total transfected plasmids. For mobility shift assay in Fig. 3E and Fig. S3, S2 cells were cotransfected with 0.8µg of pAc-*Clk*-V5 in combination with 0.6µg pMT-*ck1α*(WT)-FH, pMT-*ck1α*(K49R)-FH, or pMT-FH. 36 hours following transfection, kinase expression was induced with 500 µM $CuSO_4$ for 24 hours and treated with cycloheximide (CHX) (Sigma-Aldrich) (10µg/ml) and MG132 (Sigma-Aldrich) (25µg/ml) for 4 hours. For CLK(S13) phosphorylation detection in Fig. 3H, cells were transfected with 0.8µg of pAc-*Clk* (WT or S13A)-V5-His with either 0.6µg of pMT-*ck1α*-FH or pMT-FH. 24 hours following transfection, kinase expression was induced with 500 µM $CuSO_4$ for 24 hours. For CLK(S13) phosphorylation detection in Fig. 7D, cells were transfected with 0.8µg of pMT-FH-*Clk*(WT), 0.1µg of pMT-*dbt*(K/R), 0.1µg of pMT-*ck1α*-cmyc and pAc-*per*(WT or Δ)-NLS-V5 and induced with 500 µM $CuSO_4$ immediately after transfection. For CHX chase assay in Fig. S4, S2 cells were transfected with 0.8µg of pAc-*Clk*(WT)-V5 and either 0.6µg of pMT-*ck1α*-FH or pMT-FH.

For luciferase reporter assay in Fig. 2C and Fig. 3A, S2 cells were cotransfected with the plasmid combination as indicated: 0.025µg of *per-luc*, 0.025µg of *ren-luc* and 0.002µg of pAc-*Clk*(X)-V5 where X is either WT, S13A, S13D or cold isoform. S2 cells were harvested 36 hours after transfection prior to reporter assay. For luciferase reporter assay in Fig. 7, S2 cells were cotransfected with the plasmid combination as indicated: 0.1µg of *per-E-box-Luciferase*, 0.1µg of pCopia *Renilla-luciferase*, 8ng of pAc-*Clk*(WT)-V5, 8ng of pMT-*ck1α*-FH and 80ng of pAc-*per*(WT)-V5 or pAc-*per*(WT or Δ)-NLS-V5. Kinase expression was induced with 500 µM $CuSO_4$ immediately after transfection and cells were harvested 44 hours after induction.

Luciferase reporter assay

Measurements were performed using the Dual-Glo Luciferase Assay System following the instructions of manufacturer (Promega). Two technical replicates were performed for each biological luciferase reporter assay replicates. Three to four biological replicates were performed.

Chromatin Immunoprecipitation (ChIP)

CLK-ChIP was performed as described previously[9]. All buffers described below, except ChIP Elution buffer, contain 1X SIGMAFAST EDTA-free protease inhibitor and 0.5 mM PMSF. Briefly, fly head tissues were homogenized using liquid nitrogen chilled mortar and pestle, mixed with Nuclear Extraction buffer (NEB) (10mM Tris-HCl pH 8.0, 0.1mM EGTA pH 8.0, 10mM NaCl, 0.5mM EDTA pH 8.0, 1mM DTT, 0.5% Tergitol NP-10, 0.5mM Spermidine, 0.15mM Spermine), and lysed with a glass dounce homogenizer (Wheaton, Millville, NJ). Homogenate was transferred to a 70μm cell strainer (Thermo Fisher Scientific) prior to centrifugation at 300 g for 1 minute at 4°C. Supernatant were centrifuged at 6700 rpm for 10 minutes at 4˚C. Pellets were resuspended in NEB buffer prior to centrifugation at 11,500 rpm for 20 minutes at 4˚C on a sucrose gradient (1.6M sucrose in NEB and 0.8M sucrose in NEB). Nuclei-containing pellets were fixed with 0.3% formaldehyde in NEB and rotated at room temperature for 10 minutes. Glycine was then added at a final concentration of 0.13mM to quench crosslinking. Samples were centrifuged at 6,500 rpm for 5 minutes at 4˚C. Pellets (cross-linked chromatin) were washed twice with NEB and resuspended in Sonication buffer (10mM Tris-HCl pH 7.5, 2mM EDTA pH 8.0, 1% SDS, 0.2% Triton X-100, 0.5mM Spermidine, 0.15mM Spermine). The cross-linked chromatin was sheared by sonicator (Q80023, QSonica, Newton, Connecticut) to roughly 500 base pair fragments. Supernatant (sheared chromatin) was collected after the centrifugation at 10,000 rpm for 10 minutes. 1.5μl of CLK antibodies[10] were incubated with 25μl of Dynabeads (Thermo Fisher Scientific) in ChIP Wash buffer (50mM Tris-HCl, 1mM EDTA pH 8.0, 1% Triton X-100, 0.1% DOC, 10μg/ml AcBSA (Promega), 100mM KCl in 1X PBS, 150mM NaCl, 5mM EGTA pH 8.0, 0.1% SDS) at 4˚C for 2 hours. Following incubation, beads were collected using a magnet stand (Sigma-Aldrich) and incubated with sheared chromatin that were diluted 10-fold with IP buffer (50mM Tris-HCl pH 7.5, 2mM EDTA pH 8.0, 1% Triton X-100, 0.1%

DOC, 150mM NaCl, 0.5mM EGTA pH 8.0) at 4˚C for 2 hours. Beads were then collected and washed twice with ChIP Wash buffer for 30 minutes at 4°C, once with LiCl Wash buffer (10mM Tris-HCl pH 8.0, 250mM LiCl, 0.5% NP-40, 0.5% DOC, 1mM EDTA pH 8.0) for 30 minutes at 4˚C and once with TE buffer (1mM EDTA pH 8.0, 10mM Tris-HCl pH 8.0) for 4 minutes at 4˚C. Beads were eluted with ChIP Elution buffer (50mM Tris-HCl pH 8.0, 10mM EDTA pH 8.0, 1% SDS, 1mM DTT, 50mM NaCl, 4U/ml Proteinase K (NEB, Ipswich, MA), 50μg/ml RNase A (Thermo Fisher Scientific) at 37˚C for 2 hours and de-crosslinked at 65˚C overnight. Finally, DNA was purified by QIAquick PCR Purification Kit (Qiagen) and quantified by real-time qPCR. Primers for the *per CRS*, *tim E-box* were described previously[9]. Primers for the *vri E-box* are in Table S5. The average of ChIP signals for two intergenic regions, one on chromosome 2R (see Table S5) and one on the X chromosome[9], was used for non-specific background deduction unless otherwise noted. Three technical replicates were performed for each biological ChIP replicate. At least three biological ChIP replicates were performed.

Coimmunoprecipitation experiments in *Drosophila* S2 cells

CoIP experiments were performed as described previously[8] with the following modifications. *Drosophila* S2 cells were harvested 40 hours after transfection, washed once with 1X PBS and lysed with modified RIPA (20mM Tris-HCl pH 7.5, 150mM NaCl, 10% glycerol, 1% Triton X-100, 0.4% sodium deoxycholate, 0.1% SDS) supplemented with 1mM EDTA pH 8.0, 25mM NaF, 0.5mM PMSF, and SIGMAFAST EDTA-free Protease inhibitor tablet (Sigma-Aldrich). Proteins were incubated with 20μl α-V5 or α-FLAG M2 resins (Sigma-Aldrich) for 4 hours at 4˚C to pull down CLK or CK1α, respectively. Resins were washed three times in 500μl modified RIPA buffer at 4˚C using end-over-end rotator. Immune complexes were analyzed by Western blotting. Signal intensity of interacting protein was normalized to the intensity of the bait protein. Three biological replicates were performed.

Western blotting and antibodies

Western blotting and image analysis were performed as previously described[9]. Upon extraction, protein concentration was measured using Pierce Coomassie Plus Assay Reagents (Thermo Fisher Scientific). 2X SDS sample buffer was added and the mixture boiled at 95˚C for 5 minutes. Equal

amounts of proteins were resolved by polyacrylamide-SDS gel electrophoresis (PAGE) and transferred to nitrocellulose membrane (Bio-Rad) using Semi-Dry Transfer Cell (Bio-Rad). Membranes were incubated in 5% Blocking Buffer (Bio-Rad) for 40 minutes, incubated with primary antibodies for 16-20 hours. Blots were then washed with 1X TBST for 1 hour, incubated with secondary antibodies for 1 hour, and washed again prior to treatment of Clarity chemiluminescence ECL reagent (Bio-Rad). The following percentage of polyacrylamide-SDS gel were used: 8% for CLK and PER, 10% for HSP70, and 12% for CK1α.

Primary antibodies: α-CLK at 1:2000, α-pS13 at 1:1000, α-V5 (Thermo Fisher Scientific) at 1:1000 for CLK-V5, α-cmyc (Sigma-Aldrich) at 1:2000 for CK1α-cmyc, α-FLAG (Sigma-Aldrich) at 1:7000 for CK1α-FLAG and α-HSP70 (Sigma-Aldrich) at 1:10000. Secondary antibodies conjugated with HRP were added as follows: α-guinea pig IgG (Sigma-Aldrich) at 1:2000 for α-CLK, α-rabbit IgG (GE Healthcare) at 1:1000 for α-pS13, α-mouse IgG (Sigma-Aldrich) at 1:1000 for α-V5 detection, 1:2000 for α-c-myc detection, 1:2000 for α-FLAG detection and 1:10000 for α-HSP70 detection.

Phos-Tag gel electrophoresis and Western blotting

Drosophila S2 cells were lysed with extraction buffer 2 (EB2) (20mM Hepes pH 7.5, 100mM KCl, 5% glycerol, 1mM DTT, 0.1% Triton X-100, 25mM NaF, 0.5mM PMSF, 10 µg/ml Aprotinin, 5µg/ml Leupeptin, 1µg/ml Pepstatin A) supplemented with 1X PhosSTOP (Roche, Palo Alto, CA). Protein extracts were resolved using 5% SDS-PAGE at 150V for 4 hours. Resolving gel (2.13ml autoclaved water, 0.83ml 30%(w/v) acrylamide solution (30% T, 3.3% C), 1.875ml 1M Tris-HCl pH 8.8, 50µl 10mM $MnCl_2$, 50µl 10% SDS, 5µl TEMED, 7.5µl 25% APS) was cast with 10µM of Phos-Tag (Wako, Richmond, VA), followed by stacking gel (1.7ml autoclaved water, 360µl 30%(w/v) acrylamide solution (30% T, 3.3% C), 300µl 1M Tris-HCl pH 6.8, 24µl 10% SDS, 5µl TEMED, 7.5µl 25% APS). Protein ladder (Precision Plus Protein Dual Color Standards, Bio-Rad) was treated with 1M $MnCl_2$ at 1:80 prior to SDS-PAGE. 2X SDS sample buffer without EDTA (100mM Tris-HCl (pH 6.8), 4% SDS, 10% β-Mercaptoethanol, 20% glycerol, 0.01% (w/v) bromophenol blue) was used. Once resolved, gels were incubated for 10 minutes with gentle agitation first in transfer buffer (48mM Tris, 39mM Glycine, 20% Methanol, 0.000375% SDS) containing 1mM EDTA pH 8.0 followed by transfer buffer without EDTA. Proteins were then transferred onto PVDF membranes (Bio-Rad) and visualized by Western blotting. α-V5 (Thermo Fisher Scientific) at 1:1000 in combination

with α-mouse IgG (Sigma-Aldrich) at 1:1000 was used for detection of CLK-V5. Three biological replicates were performed.

Identification of CLK phosphorylation sites from *Drosophila* S2 cells

To generate stable *Drosophila* S2 cell lines for the identification of CLK phosphorylation sites, 1µg of pMT-FH-*Clk* and 1µg of pMT-*ck1α*(WT)-6Xc-myc or pMT-*ck1α*(K49R)-6Xc-myc in combination with 1µg of pCoHygro plasmid expressing hygromycin resistance were used for transfection using Effectene (Qiagen). *ck1α*(K49R)-6Xc-myc encodes a kinase dead variant of the kinase. Stable cell lines were established by selection with Schneider's *Drosophila* medium supplemented with 300µg/ml hygromycin (Roche).

Drosophila S2 cells were harvested by centrifuging at 4,000 rpm for 10 minutes at 4˚C. Supernatant was removed and then the cell pellet was washed once with 15ml of 50mM Hepes (pH 7.6). Cells were homogenized in lysis buffer (20mM Hepes pH 7.6, 5% glycerol, 350mM NaCl, 0.1% Triton X-100, 1mM DTT, 1mM $MgCl_2$, 0.5mM EDTA pH 8.0, 25mM NaF), supplemented with Complete EDTA-free Protease inhibitor cocktail (Sigma-Aldrich), and PhosSTOP (Roche), by using a 40 ml loose dounce homogenizer (Wheaton). Lysed cells were nutated at 4˚C for 30 minutes and then centrifuged at 15,000 rpm for 15 minutes at 4˚C. Immunoprecipitation was performed at 4˚C overnight with 120µl α-FLAG M2 beads (Sigma-Aldrich) followed by two 10-minute washes using lysis buffer without EDTA, DTT, and PhosSTOP. Bound proteins were eluted with equal bead volume (120µl) of elution buffer (30% glycerol, 3% SDS, 6 mM EDTA pH 8.0, 150 mM Tris pH 6.8) at 95˚C for 4 minutes. Eluted proteins were then reduced with 20 mM DTT at 65˚C for 20 minutes followed by alkylation at room temperature for 20 minutes with 100 mM Ioacetamide. Proteins were then analyzed by Coomassie staining on a 12% SDS-PAGE gel and CLK containing band was excised for mass spectrometry analysis as described in Chiu et al.[11].

Maxquant and Skyline analysis

Mass spectrometric data were processed with MaxQuant[12] version 1.6.1.0. MS/MS spectra were searched against the complete Uniprot *Drosophila melanogaster* protein database using the built-in

Andromeda peptide search engine[13] with trypsin designated as the digestion enzyme and two missed cleavages were allowed. Oxidation, N-terminal acetylation, phosphorylation, and deamination of asparagine and glutamine were selected as variable modifications. Carbamidomethylation of cysteine was selected as fixed modification. For all other parameters, MaxQuant default values were selected. Briefly, peptide tolerance for the initial and main search of Andromeda were specified at 20 ppm and 4.5 ppm respectively. For identification, an FDR of 0.01 was selected for peptide spectrum matches (PSM) and protein matches. MaxQuant output data was further processed using Skyline[14] version 4.1.0.11796. For spectral library building, a cut-off score of 0.95 was selected. For MS1 filtering, precursor with charges of 2, 3, and 4 were considered. For retention time filtering, only scans within 5 minutes of MS/MS identification were selected. Quantification of phosphorylated peptides were performed as area under the curve of each identified peptide.

For quantification of relative phosphopeptide abundance, chromatograms of peptides, shown in [15], were extracted using Skyline version 4.1.0.11796.

Generating CLK(S13) phosphospecific antibodies

Phosphospecific antibodies were generated by PhosphoSolutions (Denver, CO). Rabbits were immunized with a 15-amino-acid peptide (amino acid 6-DDKDDTKpSFLCRKSR-amino acid 20); where pS = phosphoserine). The resulting rabbit sera was further affinity-purified using the pS13 phosphopeptide.

Detection of CLK pS13

Proteins from S2 cells were extracted using EB2 (20mM HEPES pH 7.5, 100mM KCl, 5% Glycerol, 5mM EDTA, 0.1% Triton X-100, 0.5mM PMSF, 1mM DTT, 10 mg/ml Aprotinin, 5 mg/ml Leupeptin, 1 mg/ml Pepstatin) supplemented with 1X PhosSTOP (Roche) and 25mM NaF. Immunoprecipitation to enrich for CLK proteins was performed as described previously[11] using 20 μl of α-V5 resin per IP reaction. CLK pS13 is then detected by Western blots using CLK(pS13) phosphospecific antibody. Fly protein was extracted using RBS (20mM HEPES pH7.5, 50mM KCl, 10% glycerol, 2mM EDTA, 1mM DTT, 1% Triton X-100, 0.4% NP-40, 10 mg/ml Aprotinin, 5 mg/ml Leupeptin, 1 mg/ml

Pepstatin, 0.5mM PMSF, 25mM NaF, 1X PhosSTOP(Roche)) and analyzed by Western blotting in combination with CLK(pS13) phosphospecific antibody directly without IP.

Locomotor activity assay

Daily locomotor activity rhythms in male flies were assayed using the *Drosophila* Activity Monitoring System (DAMS, TriKinetics, Waltham, MA) as described previously[16].

Immunofluorescence and confocal imaging

Brain dissections and immunofluorescence staining procedures were performed as described previously[10]. 3-5-day old flies were entrained for 4 days in 12h:12h LD and fixed with 4% paraformaldehyde for 40 minutes at ZT3 and ZT15 on LD4. Brains were washed three times in 1XPBST (0.1% Triton X-100 in PBS), blocked with 10% Normal Goat Serum (Jackson Immunoresearch, West Grove, PA) in PBST for 90 minutes and incubated with primary antibodies two nights. Primary antibodies against PDF (C7-C; Developmental Studies Hybridoma Bank, Iowa City, IA) was used at 1:1000. Brains were then washed and probed with secondary antibodies α-mouse IgG Alexa Fluor 647 (Jackson Immunoresearch, 115-605-003) at 1:1000. Nine to ten fly brains for each genotype at each time-points were dissected and imaged. Representative images are shown. Fiji software[17] was used for image analysis.

Cycloheximide (CHX) chase assay

24 hours following S2 cell transfection, *ck1α* expression was induced for 16 hours prior to treatment with CHX to stop protein synthesis (10µg/ml) (Sigma-Aldrich). Cells were then harvested and lysed with EB2 supplemented with 5mM EDTA pH 8.0 at the times indicated after CHX addition. Protein lysates were analyzed by Western blotting.

Cloning, expression and purification of CLK-bHLH

Unless otherwise specified, LB Broth (Lennox) (Sigma-Aldrich) was used as the culturing media for *Escherichia coli*. Cloning was performed in DH5α cells (Thermo Fisher Scientific). The coding

sequence corresponding to the bHLH domain of *Drosophila* CLK (aa 1-71) was PCR amplified from pAc-*Clk*(WT)-V5 (Kim and Edery, 2006) and introduced into the expression vector pET22b using the restriction sites NdeI and SalI (New England Biolabs). A His_6-tag-STOP sequence was introduced into the SalI-reverse primer to enable affinity purification via immobilized metal affinity chromatography (IMAC). S13D mutagenesis was performed using mutagenic primers and Pfu Turbo Cx DNA polymerase (Agilent Technologies, Santa Clara, CA) (See Table S5 for mutagenic primer sequences).

For protein expression and purification, BL21(DE3) (Thermo Fisher Scientific) was transformed with the resultant plasmids, pET22b-CLK71-His_6 and pET22b-CLK71(S13D)-His_6. Single colonies were picked from each clone and inoculated into 25-ml starter cultures and grown at 37°C until OD_{600} ~ 0.5 was reached. Then, the cultures were diluted at a 1:100 ratio in 2 500-ml expression cultures each (1L per construct) and grown at 30°C until OD_{600} ~ 0.5 was reached. Cultures were then transferred to 4°C for 30 minutes, induced with 1 mM isopropyl-β-D-1-thiogalactopyranoside (IPTG), supplemented with 2% glycerol (final concentrations), and grown at 18°C for 16 hours. Cells were harvested by centrifuging at 4°C at 4,000 rpm for 15 minutes (Sorvall) and resuspended in 40 ml lysis buffer (50 mM sodium phosphate, 500 mM NaCl, 10 mM β-mercaptoethanol, pH 8.0) per construct. The resuspended cells were sonicated over ice with a sonicator (Sonic Dismembrator Model E150E, Thermo Fisher Scientific) at 90% amplitude for 10 cycles of 1-min on and 1-min off. The lysates were then treated with 10 µl DNase I (New England Biolabs) and incubated at 4°C for 1 hour on a rotator followed by centrifugation at 13,000 rpm, 4°C for 30 minutes to remove cell debris. The clarified lysates were then filtered through 0.22 µm filters (EMD Millipore, Burlington, MA) and applied onto an nickel(II)-nitrilotriacetic acid (Ni-NTA) IMAC column (Bio-Rad) on a chromatography platform (NGC, Bio-Rad), washed with 82.5 mM imidazole and eluted on a 82.5 mM – 250 mM imidazole gradient. Fractions containing CLK71-His_6 or CLK71(S13D)-His_6 were pooled and dialyzed to working buffer (1× PBS supplemented with 10 mM β-mercaptoethanol) using dialysis cassettes (3.5K MWCO, Thermo Fisher Scientific). Concentrations were measured using Coomassie Plus reagent (Thermo Fisher Scientific) using bovine serum albumin (BSA) (Thermo Fisher Scientific) as a standard. The purified constructs were aliquoted, frozen in liquid nitrogen, and stored at −80°C until further use.

Size exclusion chromatography

CLK71-His_6 or CLK71(S13D)-His_6 constructs were diluted in working buffer to 50 μM. 250 μL of the diluted proteins were loaded onto a Bio-Rad ENrich 70 10 x 300 column and eluted with working buffer. The column was pre-calibrated with 250 μL of 5-fold diluted gel filtration standard (Bio-Rad) and fitting the peak positions of chicken ovalbumin (44 kDa), equine myoglobin (17 kDa), and vitamin B12 (1350 Da), to the formula $\log_{10} M_w = aV + b$, where *a* and *b* were empirically determined. The molecular weight of the CLK constructs in solution were then estimated from their peak positions in their chromatograms.

Biolayer Interferometry (BLI) data acquisition

BLI was performed using the Octet RED384 system (Sartorius, Göttingen, Germany). All steps were performed in Kinetics buffer (1X PBS, 0.02% Tween-20, 0.1% BSA, 0.05% sodium azide, pH 7.4, Sartorius) unless otherwise stated. For the BLI bait, the 21-bp *per* promoter (5'-CCGCCGCTCACGTGGCGAACT-3') and scrambled (5'-GTACGCTGCAGGCCCCCTGAC-3', identical GC content) DNA were purchased from IDT (San Diego, CA). In both cases, the forward oligomers were purchased as 5' biotin conjugates and annealed in-house with their respective unlabeled complementary strands (1 nmol each) at 95°C for 2 minutes and allowed to cool down to room temperature in a heat block, after which both were diluted to 200 nM in autoclaved water. Nonspecific binding between biosensors and the CLK constructs independent of DNA binding were quantified by functionalizing the Octet Streptavidin (SA) biosensors with biotin (200 nM) only.

For the CLK71-His_6 or CLK71(S13D)-His_6 constructs, 675 μL of 20 μM proteins in Kinetics buffer were prepared by diluting the dialyzed proteins in PBS and supplementing with 10X Kinetics buffer. Then, serial dilutions were performed at a 2:1 dilution ratio for 13 points, resulting in a protein concentration range of 20 μM to 103 nM. Additional wells were filled with Kinetics buffer to account for baseline drift. To obtain BLI binding kinetics, SA Biosensors (Sartorius) were treated as follows: (1) preconditioning/soaking (60s), (2) biotin-DNA functionalization in autoclaved water (200s), (3) baseline (180s), and (4) binding (300s). Data were collected at a sampling frequency of 5 Hz (0.2 s intervals).

BLI data analysis

The raw BLI kinetic trace data were preprocessed on OctetAnalysis (Sartorius) and then exported to MATLAB (Natick, MA) for further analysis using custom codes. The response arising from CLK-DNA binding, ΔΔR, was computed by subtracting individual kinetic traces by means of double subtraction of (i) traces generated from biosensors functionalized with biotin only and (ii) traces generated from DNA-coated biosensors in the absence of CLK-bHLH, as implemented in OctetAnalysis. As we observed negative response during initial binding (t = 0.2 s) for some ΔΔR traces when the biosensors were switched between baseline and binding steps after default preprocessing, indicating incomplete inter-step correction of optical artifacts and/or slight changes in buffer composition, we performed additional custom preprocessing in MATLAB by adjusting the baseline for the binding such that the responses would reach exactly 0 nm when extrapolated to t = 0 s. Then, the quasi-steady state response, defined as the average ΔΔR in the time window t = 290 – 300 s or the last 10 seconds of the binding step, was plotted against initial CLK concentration $[\mathrm{CLK}]_0$ and fitted to a 4-parameter Hill equation model,

$$R_{\mathrm{Hill}} = R_0 + \frac{R_{max}}{1 + \left(\frac{\mathrm{EC}_{50}}{[\mathrm{CLK}]_0}\right)^{n_{Hill}}} + \varepsilon$$

where R_0 is the basal response, R_{max} is the maximum change in response elicited by binding, EC_{50} is the half maximal effective concentration, n_{Hill} is the Hill coefficient, and ε is the error term. To determine if the data could be explained by nonspecific interactions alone, we also fitted the same data to the noise model,

$$R_{\mathrm{noise}} = R_0 + \varepsilon$$

The model-dependent parameters were fitted for each CLK construct-DNA bait pair using the MATLAB function `fitnlm` with R_{max}, EC_{50}, and n_{Hill} constrained to nonnegative values via logarithmic transform. The 95% confidence intervals were estimated by the Wald method (function `coefCI`). The corrected Akaike information criteria, AICc, was used to determine whether the data was better explained by specific (Hill model) or nonspecific (Noise model) binding.

Statistical analysis

RAIN[18] and CircaCompare[19] test were performed in R. Other statistical analyses were performed using GraphPad Prism 10.0 (GraphPad Software, La Jolla, California). Two-tailed Student's t test were performed if only two groups were compared. ANOVA was performed if there are more than two groups compared. One-Way ANOVA and Dunnett post hoc test were chosen if there are one independent variables, whereas Two-Way ANOVA and Šídák's post hoc test were chosen if there are two independent variables. Asterisks indicate significant differences in mean values between genotypes or conditions at indicated time-points.

SI Figures

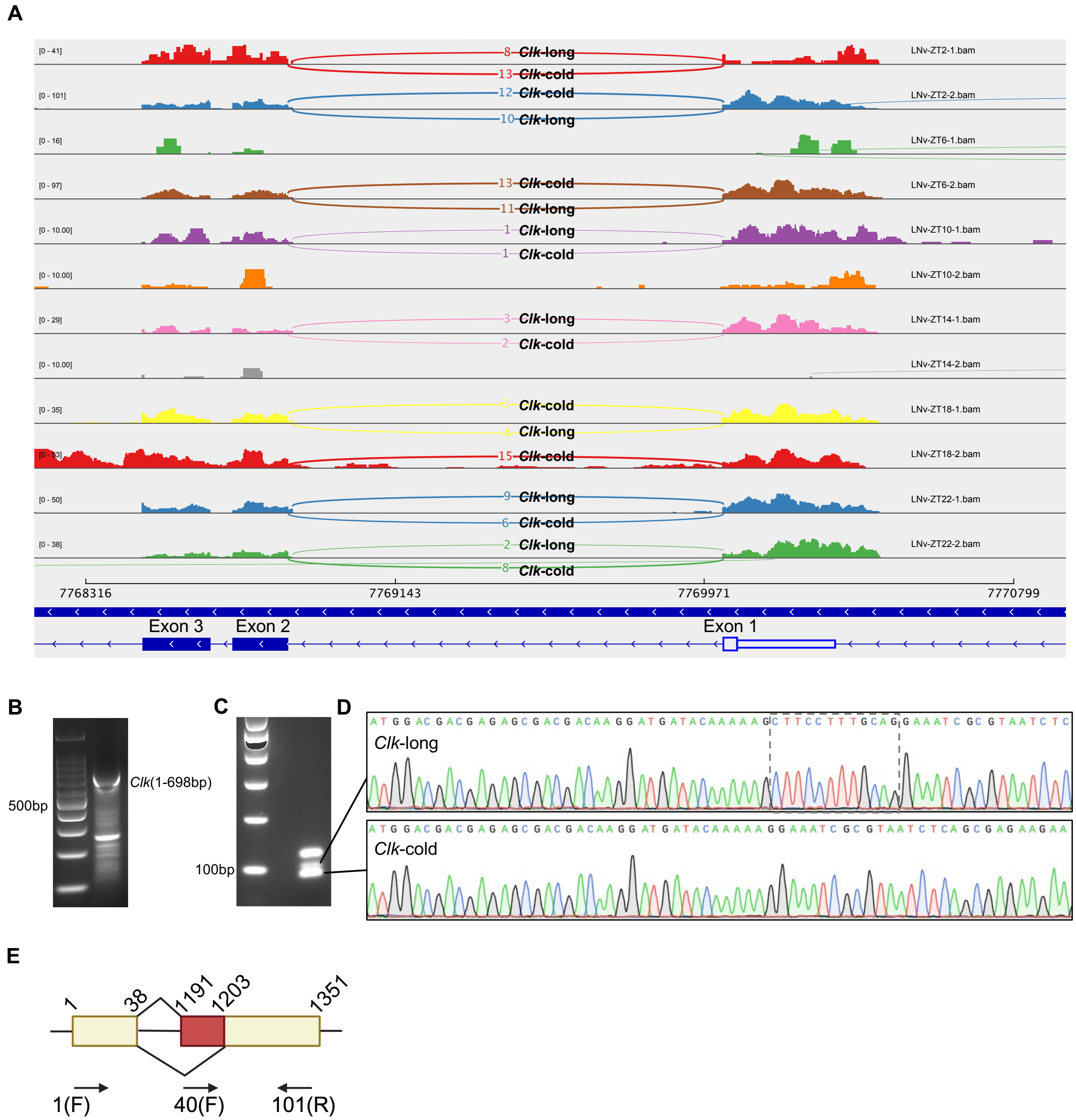

Figure S1. Expression of *Clk*-cold isoform in heads of w^{1118} flies. A. RNA-seq data tracks from Wang et al[20]. are shown to illustrate alternative splicing of *Clk* transcripts at exon 2 in small LNv_s circadian neurons, with arcs showing splice junctions and the number of unique-mapped RNA-seq reads mapped to the junction across the arc. The orientation of the transcript is indicated by blue arrows at the bottom. **B.** Agarose gel showing reverse transcription product from heads of w^{1118} flies collected at ZT0 on LD3

using *Clk*(698R) gene-specific primer, amplified by PCR using *Clk*(1F) and *Clk*(698R) primers (see Table S5 for sequences of primers). **C.** Agarose gel showing *Clk*-long and *Clk*-cold isoforms amplified by PCR using *Clk*(1F)-*Clk*(101R) primers with gel extract from **B**. The top band was determined to be a hybrid of the two isoforms, likely a PCR artifact. **D.** Chromatogram that confirms the isolation of each isoform. **E.** Schematic representation of primers to measure the relative levels of two *Clk* transcripts that vary at the alternative 3' splice site at exon 2. Exon-intron organization of *Clk* and the relative positions of the three primers used in this study for nested RT-qPCR. Two *Clk* transcripts quantified by qPCR via either retention (*Clk*-long) or removal (*Clk*-cold) of the 12bp of exon 2 (indicated in red).

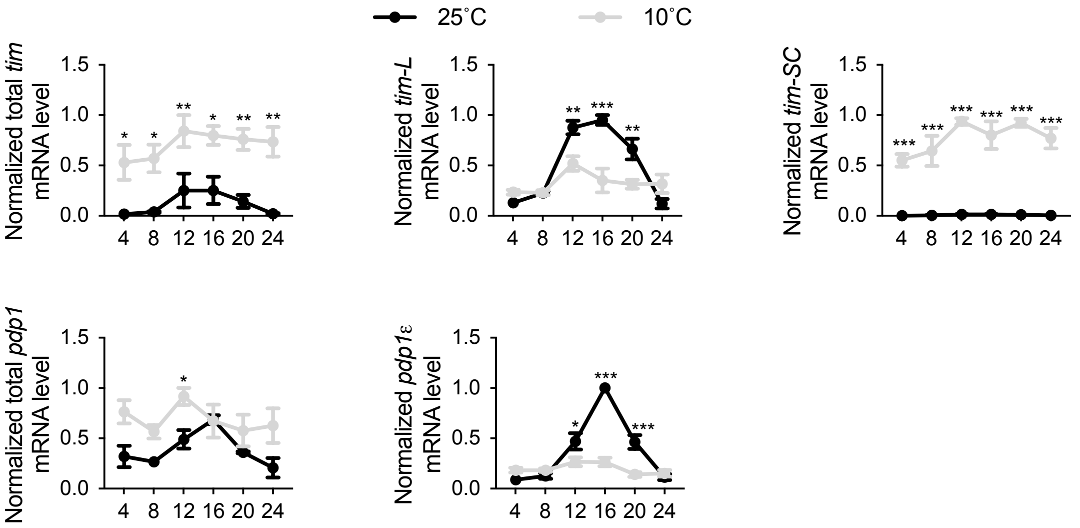

Figure S2. Cold temperature promotes mRNA expression of CLK targets. Daily steady state mRNA expression of CLK targets (*tim* and *pdp1*) in heads of w^{1118} flies. *tim*-L, *tim*-SC, *pdp1ε* isoforms were analyzed with isoform-specific primers. Flies were entrained in 12h:12h LD and collected on LD3 at the indicated temperatures and time-points (ZT) (n=3). Error bars indicate ± SEM, ***p<0.001, **p< 0.01, *p< 0.05, Two-Way ANOVA and Šídák's post hoc test.

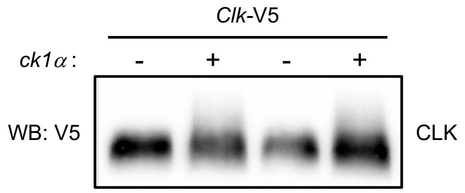

Figure S3. CK1α induces detectable but mild mobility shift of CLK on regular SDS-PAGE gel. *Drosophila* S2 cells were transfected with pAc-*Clk*-V5 in combination with either pMT-*ck1α*-3XFLAG-6XHIS or pMT-3XFLAG-6XHIS empty plasmid. Protein extracts were analyzed on regular SDS-PAGE gel followed by western blotting with α-V5. Blots of two biological replicates were shown on the same gel.

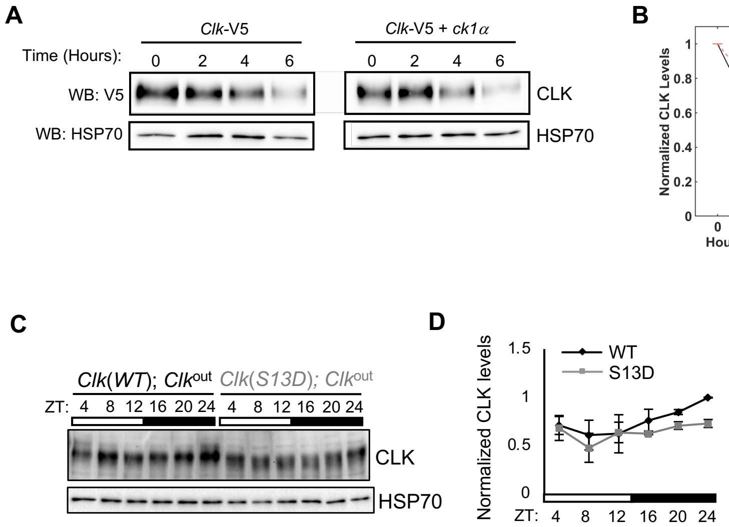

Figure S4. CK1α does not regulate CLK stability. A. *Drosophila* S2 cells were cotransfected with pAc-*Clk*-V5-His in combination with either pMT-*ck1α*-FH or pMT-FH empty plasmid. Following a 24-hour incubation period, kinase expression was induced with $CuSO_4$. Cycloheximide (CHX) was added 16 hours after kinase induction and cells were harvested for protein extractions at the indicated times after addition of CHX. Proteins were visualized by Western blotting and detected with α-V5. α-HSP70 was used to indicate equal loading and for normalization. **B.** Quantification of CLK in **A**. Error bars indicate ± S.E.M (n=2). **C.** Western blots comparing CLK protein profiles in heads of *Clk*(WT) and *Clk*(S13D) entrained in 12h:12h LD at 25˚C and collected at indicated time-points on LD3. α-HSP70 was used to indicate equal loading and for normalization. **D.** Quantification of CLK in **C** (n=3). Error bars indicate ± SEM, **p< 0.01, Two-Way ANOVA and Šídák's post hoc test.

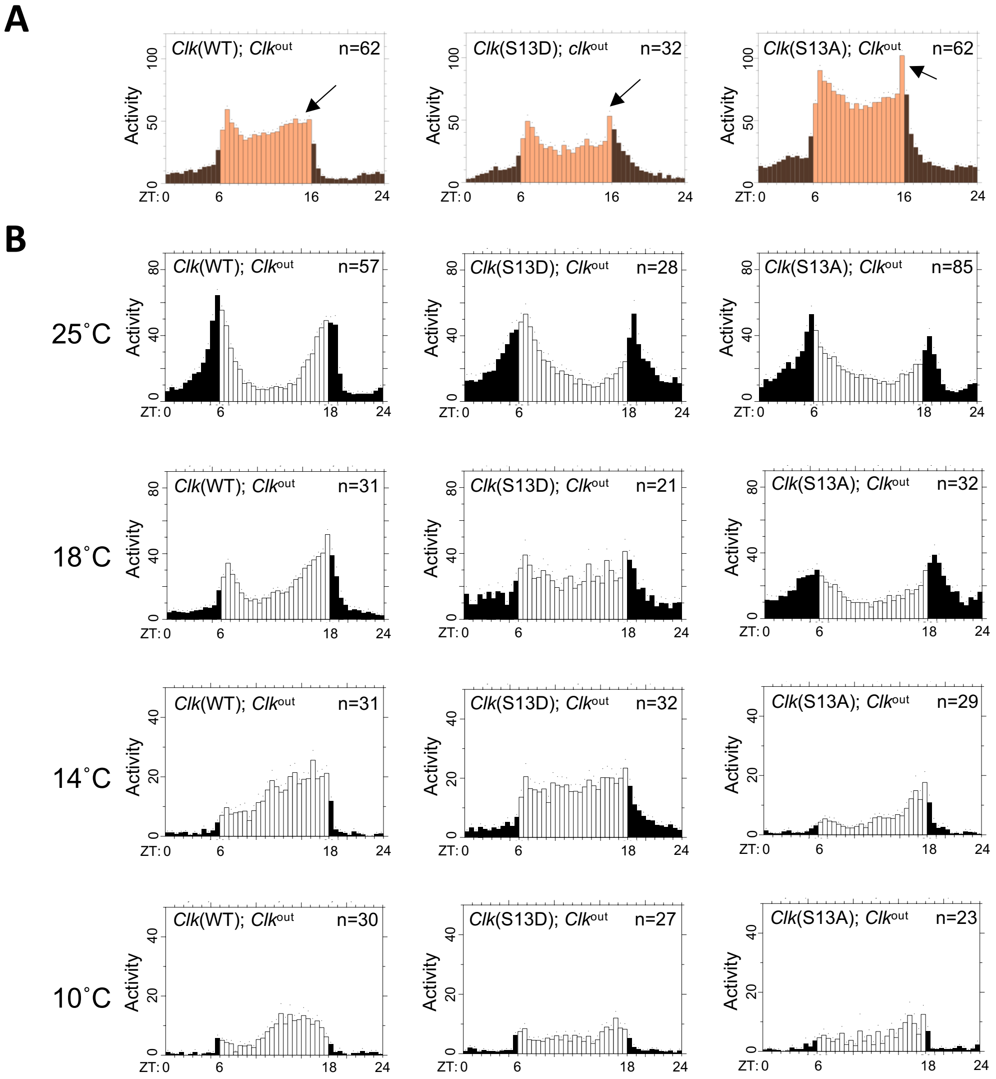

Figure S5. Flies expressing CLK(S13) variants display anticipation under thermic and photic entrainment. Eduction graphs showing the average activity in day one through day four. Flies were entrained in (A) 10h:14h 25˚C/17˚C temperature cycles at constant darkness (DD) for four days and (B) 12h:12h LD at indicated temperatures. Average activity of each genotype was plotted using FaasX. n represents the sample size. Arrows in (A) indicate anticipatory activity prior to evening cryophase.

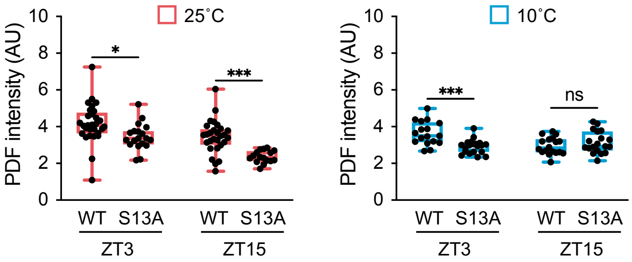

Figure S6. *Clk*(S13A) mutants display altered PDF intensity. Quantification of PDF intensity in dorsal projection of sLNvs neurons in adult fly brains stained with α-PDF (C7). Flies were entrained for 4 days in 12h:12h LD cycles and collected at the indicated times and temperature on LD4 for fixation and immunofluorescence analysis. Error bars indicate min to max, *p<0.05, **p< 0.01, ***p<0.001, Two-Way ANOVA and Šídák's post hoc test. *This is the same data from Figure 5C, but plotted differently to highlight the difference between Clk(WT) and Clk(S13A) flies.*

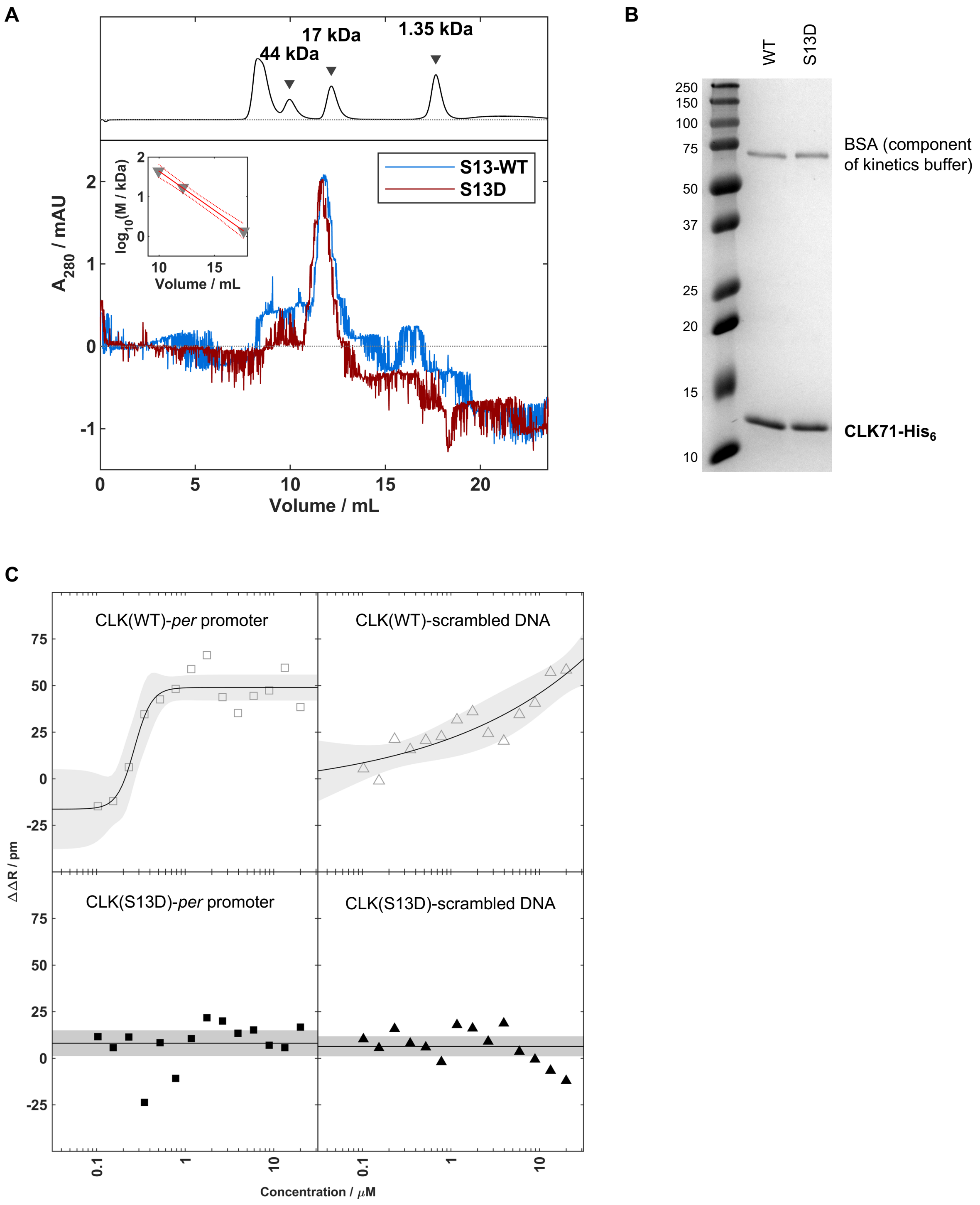

Figure S7. Additional characterization of CLK-bHLH constructs. A. Size exclusion chromatography of CLK71-His6 constructs suggests that both constructs are homodimeric (S13-WT, blue, 19.7 kDa, 95% CI = [15.3, 27.1]; S13D, dark red, 20.4, 95% CI = [15.3, 27.1]) assuming globular structure. The calibration

standard with the peaks of chicken ovalbumin (44 kDa), equine myoglobin (17 kDa), and vitamin B12 (1350 Da) are shown above, whereas the standard curve is shown in the inset. **B.** Coomassie-stained 16% SDS-PAGE of constructs in 1X Kinetics buffer (containing 0.1% BSA) used in Biolayer Interferometry. **C**. Quasi-steady state signal response of CLK-bHLH-DNA binding in the presence (black, filled) and absence (grey, hollow) of the phosphomimetic S13D mutation are plotted separately for the 21-bp *per* promoter DNA sequence (squares) or a scrambled sequence with identical GC content (triangles). Solid lines and shaded areas show fits and 95% prediction interval to the 4-parameter Hill equation for CLK-bHLH-WT and nonbinding baseline for CLK-bHLH-S13D.

SI Tables

Table S1. Potential kinases that phosphorylate CLK(S13) predicted by GPS5.0[21]

Kinase	Score[a]	Cutoff[b]
CK1	8.572	6.306
AGC/GRK	4.082	2.938
AGC/PKC	-2.713	-3.397
CK1/CK1	8.982	7.443
CMGC/CLK	108.406	85.655
TKL/LRRK	15.219	13.084
Other/Haspin	5.068	0.998
Other/MOS	3.312	2.885
Other/NKF2	16.23	15.808
Other/PLK	15.194	14.476
Other/TLK	3.196	2.277
Other/TOPK	4	3.19
AGC/Akt/AKT1	12.108	11.65
AGC/DMPK/CRIK	2.113	0.919
AGC/GRK/GRK	16.967	13.902
AGC/PKC/PKCa	10.105	9.03
AGC/PKC/PKCh	10.857	10.374
AGC/PKC/PKCi	6.637	3.394
CAMK/CAMKL/NuaK	5.463	5.434
CAMK/DAPK/DRAK	8.733	4.561
CAMK/MAPKAPK/MNK	25.53	18.111
CAMK/PIM/PIM1	0.001	0.001
CAMK/PKD/PRKD2	2.685	2.35
CK1/CK1/CK1-A	12.213	10.881
CK1/VRK/VRK1	0.412	0.394
CMGC/CDK/CDK7	20.48	20.158
CMGC/CLK/CLK2	0.008	0.006
TKL/LISK/LIMK	9.722	-0.331
TKL/LRRK/LRRK2	19.436	17.83
TKL/MLK/ILK	28.954	21.936
TKL/STKR/STKR1	16.93	11.666
Atypical/PIKK/FRAP	9.731	9.635
Other/BUB/BUB1	2.63E-05	0.00E+00
Other/Haspin/HASPIN	5.068	0.998
Other/IKK/CHUK	241.828	223.586
Other/MOS/MOS	3.312	2.885

Other/NKF2/PINK1	16.23	15.808
Other/PLK/PLK1	13.599	13.525
Other/TLK/TLK1	4.281	3.166
Other/TOPK/PBK	4	3.19
Other/TTK/TTK	23.973	22.818
Other/WEE/Myt1	2.576	2.486
Other/WNK/WNK4	3.321	3.101
AGC/DMPK/CRIK/CIT	2.113	0.919
AGC/DMPK/GEK/DMPK	5.205	3.509
AGC/GRK/GRK/GRK1	24.441	10.931
AGC/GRK/GRK/GRK7	2.821	1.646
AGC/PKC/PKCa/PRKCA	20.009	19.505
AGC/PKC/PKCa/PRKCB	7.1	4.392
AGC/PKC/PKCh/PRKCE	18.559	18.443
AGC/PKC/PKCh/PRKCH	36.867	28.536
AGC/PKC/PKCi/PRKCI	13.68	9.121
AGC/PKC/PKCi/PRKCZ	60.502	41.793
AGC/RSK/RSKp70/RPS6KB1	32.343	30.881
CAMK/CAMKL/BRSK/BRSK2	5.087	4.325
CAMK/CAMKL/NuaK/NUAK1	5.463	5.434
CAMK/DAPK/DRAK/STK17B	6.78	3.169
CAMK/MAPKAPK/MNK/MKNK1	13.544	10.57
CAMK/MAPKAPK/MNK/MKNK2	25.807	22.762
CK1/CK1/CK1-A/CSNK1A1	26.971	24.196
CK1/CK1/CK1-D/CSNK1E	31.411	29.494
CK1/CK1/CK1-G/CSNK1G1	3.71	2.674
CMGC/DYRK/DYRK1/DYRK1B	0.167	0.087
STE/STE20/MST/STK3	85.537	73.61
STE/STE20/SLK/STK10	3.964	3.565
TKL/LISK/LIMK/LIMK1	9.722	-0.331
TKL/MLK/ILK/ILK	28.954	21.936
TKL/STKR/STKR1/ACVRL1	4.929	1.091
TKL/STKR/STKR1/BMPR1B	11.191	-1.013
Atypical/Alpha/ChaK/TRPM6	4.517	1.899
Atypical/PDHK/PDHK/PDK1	0.199	0.182
Atypical/PIKK/FRAP/MTOR	12.914	10.962
Other/PLK/PLK1/PLK1	13.751	12.929
Other/PLK/PLK2/PLK2	21.072	18.362

[a] Average similarity score between S13 containing peptide and peptides around all known phosphorylation sites.

[b] Cutoff for a predicted phosphorylation site.

Table S2. Identification of Ck1α-dependent phosphorylation sites in *Drosophila* head tissues

**Modified Residue**[a]	**Peptide Sequence**[b]	**Probability**[c]	**Higher Abundance in Presence of CK1α?**[d]
S5[e]	MDDE**S**DDKDDTKSFLCR	1	No
S13	MDDESDDKDDTK**S**FLCR	0.996	Yes
S258[f]	EM**S**IIDPTSNEFTSK	1	Yes
S311	APPIIGYMPFEVLGTSGYD YYHFDDLD**S**IVACHEELR	0.872	Yes
S487[e,f]	TSRPASSYGNISSTGI**S**PK	0.999	No
S476[f]/487[f]	TSRPA**S**SYGNISSTGI**S**PK	0.894; 0.991	Yes
T484/S487[f]	TSRPASSYGNISS**T**GI**S**PK	0.591; 0.985	Yes
S504	GND**S**DSTSMSTDSVTSR	1	No

[a] Residues are numbered according to dCLK-long (amino acids 1- 1027, UniProtKB O61735).
[b] Phosphorylated residues are bolded.
[c] Probability of phosphorylation site determined by Maxquant[12,22].
[d] Comparison of the abundance of each phosphorylated residue between co-expression of target protein with or without CK1α.
[e] Identified in Lee et al[23].
[f] Identified in Mahesh et al[1].

Table S3. Daily locomotor activity rhythms of *Clk* mutants at 25°C

Genotype	Period (h) (mean ± SEM)	Power[a]	Rhythmicity (%)[b]	No. of flies tested	No of flies surviving[c]
w; +; Clk^{out}	AR[d]	ND[e]	0	32	31
w; Clk(WT)*; Clk*^{out}	24.2 ± 0.10	68.7	86.2	63	58
w; Clk(S13D)*; Clk*^{out}	25.4 ± 0.39	50.6	39.3	32	28
w; Clk(S13A)*; Clk*^{out}	25.1 ± 0.04	56.2	60	96	85
w; Clk(WT)/+*; Clk*^{out}	24.8 ± 0.12	59.9	61.7	64	60
w; Clk(S13D)/+*; Clk*^{out}	23.5 ± 0.20	35.5	11.1	37	36
w; Clk(S13A)/+*; Clk*^{out}	25.9 ± 0.13	42.0	30.4	27	23

[a] Measures the strength or amplitude of the locomotor activity rhythm (in arbitrary units)
[b] Percentage of flies that are rhythmic
[c] Number of flies that survived until the end of the experiment
[d] AR denotes Arrhythmic
[e] ND denotes Not Determined

Table S4. Rhythmic parameters of mRNA analysis

Target	WT mesor	S13D mesor	P-value for mesor difference	WT amplitude	S13D amplitude	P-value for amplitude difference	WT peak time (ZT)	S13D peak time (ZT)	P-value for difference in phase
per	0.617	0.436	***	0.310	0.174	*	13.215	15.256	*
tim	0.521	0.208	***	0.395	0.158	***	15.743	15.906	0.755
vri	0.500	0.241	***	0.402	0.131	***	13.967	14.385	0.683
**Target**	**WT mesor**	**S13A mesor**	**P-value for mesor difference**	**WT amplitude**	**S13A amplitude**	**P-value for amplitude difference**	**WT peak time (ZT)**	**S13A peak time (ZT)**	**P-value for difference in phase**
per	0.525	0.367	*	0.390	0.173	**	14.173	12.039	0.079
tim	0.503	0.288	***	0.417	0.239	***	15.492	15.153	0.558
vri	0.496	0.220	***	0.467	0.156	***	13.895	12.350	0.091

Table S5: Primers for PCR mutagenesis, ChIP analysis and RT-qPCR analysis

Primer for mutagenesis: g*Clk*(S13A) F: AAG GAT GAT ACA AAA GCG TAA ATT CAC TAG ACA
Primer for mutagenesis: g*Clk*(S13A) R: TGT CTA GTG AAT TTA CGC TTT TGT ATC ATC CTT
Primer for mutagenesis: g*Clk*(S13D) F: AAG GAT GAT ACA AAA GAG TAA ATT CAC TAG ACA
Primer for mutagenesis: g*Clk*(S13D) R: TGT CTA GTG AAT TTA CTC TTT TGT ATC ATC CTT
Primer for mutagenesis: *Clk*(S13A) F: AAG GAT GAT ACA AAA GCG TTC CTT TGC AGG AAA
Primer for mutagenesis: *Clk*(S13A) R: TTT CCT GCA AAG GAA CGC TTT TGT ATC ATC CTT
Primer for mutagenesis: *Clk*(S13D) F: AAGGATGATACAAAAGACTTCCTTTGCAGGAAA
Primer for mutagenesis: *Clk*(S13D) R: TTTCCTGCAAAGGAAGTCTTTTGTATCATCCTT
Primer for mutagenesis: *Clk*-cold F: GGATGATACAAAAAGGAAATCGCGTAATCT
Primer for mutagenesis: *Clk*-cold R: AGATTACGCGATTTCCTTTTTGTATCATCC
Primer for ChIP: 2R intergenic (CP023338) F: TCAGCCGGCATCATTAGCAGCCG
Primer for ChIP: 2R intergenic (CP023338) R: TCGTGTGCGGGAATCTCTGCCG
Primer for ChIP: X intergenic (FBgn0003638) F: ACTGCGTATTCAGGATACATGCC
Primer for ChIP: X intergenic (FBgn0003638) R: TGTCCACTTTAATTGATTGCGTGG
Primer for ChIP: *per CRS* F: TGCCAGTGCCAGTGCGAGTTCG
Primer for ChIP: *per CRS* R: TGCCTGGTGGGCGGCTGG
Primer for ChIP: *tim E-box1* F: ACGTTGTGATTACACGTGAGCC
Primer for ChIP: *tim E-box1* R: ACACTGACCGAAACACCCAC
Primer for ChIP: *vri E-box* F: AACCAGACAGTTTGGTGGCTGGG
Primer for ChIP: *vri E-box* R: CAGTGCTAGCTAACTATTTGAACTCGTC
Primer for RT-qPCR: *per* F: GACCGAATCCCTGCTCAA
Primer for RT-qPCR: *per* R: GTGTCATTGGCGGACTTC
Primer for RT-qPCR: *tim* F: CCCTTATACCCGAGGTGGAT
Primer for RT-qPCR: *tim* R: TGATCGAGTTGCAGTGCTTC
Primer for RT-qPCR: total *tim* F: AAAAGCAGCCTCATCAACAT
Primer for RT-qPCR: total *tim* R: AGATAGCTGTAACCCTTGAG
Primer for RT-qPCR: *tim-SC* F: AACACAACCAGGAGCATAC
Primer for RT-qPCR: *tim-SC* R: ATGGTCCACAAATGTTAAAA
Primer for RT-qPCR: *vri* F: ATGAACAACGTCCGGCTATC
Primer for RT-qPCR: *vri* R: CTGCGGACTTATGGATCCTC
Primer for RT-qPCR: *pdp1ε* F: GCGGCAACTGGTAATG

Primer for RT-qPCR: *pdp1ε* R: ATTTCCTGCCTGAGCT
Primer for RT-qPCR: total *pdp1* F: TCCTCGGGCCGTGACTTTG
Primer for RT-qPCR: total *pdp1 R*: TGGCGGCAATGTTGTTCTTC
Primer for RT-qPCR: *cwo* F: TGGCGGCAATGTTGTTCTTC
Primer for RT-qPCR: *cwo* R: GTAGTTGCCCTGTCCGTGAA
Primer for RT-qPCR: *dgol* F: GCCACGGATCTATGCAGTTT
Primer for RT-qPCR: *dgol* R: CTTGGATAGCGACTGCTGTG
Primer for RT-qPCR: *Clk* F: AGGACTCGCTGTTGTCCTG
Primer for RT-qPCR: *Clk* R: TGCTGCTGCACCAGATTGC
Primer for RT-qPCR: *cry* F: CCACCGCTGACCTACCAAAT
Primer for RT-qPCR: *cry* R: GGAAGCCCATGTTGTCTCCA
Primer for RT-qPCR: *cbp20* F: GTCTGATTCGTGTGGACTGG
Primer for RT-qPCR: *cbp20* R: CAACAGTTTGCCATAACCCC
Primer for RT-qPCR: *Clk*(1F): ATGGACGACGAGAGCGACGAC
Primer for RT-qPCR: *Clk*(40F): TTCCTTTGCAGGAAATCG
Primer for RT-qPCR: *Clk*(101R): AGCGAGTTGAACTGATCTCG
Primer for RT-PCR: *Clk*(698R): TTGGGATTCTGCTGGAAGATGC

SI References

1. Mahesh, G. *et al.* Phosphorylation of the Transcription Activator CLOCK Regulates Progression through a ~24-h Feedback Loop to Influence the Circadian Period in Drosophila. *J. Biol. Chem.* **289**, 19681–19693 (2014).
2. Bischof, J., Maeda, R. K., Hediger, M., Karch, F. & Basler, K. An optimized transgenesis system for *Drosophila* using germ-line-specific φC31 integrases. *Proc. Natl. Acad. Sci.* **104**, 3312–3317 (2007).
3. Venken, K. J. T., He, Y., Hoskins, R. A. & Bellen, H. J. P[acman]: A BAC Transgenic Platform for Targeted Insertion of Large DNA Fragments in *D. melanogaster*. *Science* **314**, 1747–1751 (2006).
4. Kim, E. Y. & Edery, I. Balance between DBT/CKIepsilon kinase and protein phosphatase activities regulate phosphorylation and stability of Drosophila CLOCK protein. *Proc. Natl. Acad. Sci. U. S. A.* **103**, 6178–6183 (2006).

5. Hao, H. *et al.* The 69 bp Circadian Regulatory Sequence (CRS) Mediates *per* -Like Developmental, Spatial, and Circadian Expression and Behavioral Rescue in *Drosophila*. *J. Neurosci.* **19**, 987–994 (1999).
6. Nawathean, P. & Rosbash, M. The Doubletime and CKII Kinases Collaborate to Potentiate Drosophila PER Transcriptional Repressor Activity. *Mol. Cell* **13**, 213–223 (2004).
7. Ko, H. W., Jiang, J. & Edery, I. Role for Slimb in the degradation of Drosophila Period protein phosphorylated by Doubletime. *Nature* **420**, 673–678 (2002).
8. Lam, V. H. *et al.* CK1α Collaborates with DOUBLETIME to Regulate PERIOD Function in the *Drosophila* Circadian Clock. *J. Neurosci.* **38**, 10631–10643 (2018).
9. Kwok, R. S., Li, Y. H., Lei, A. J., Edery, I. & Chiu, J. C. The Catalytic and Non-catalytic Functions of the Brahma Chromatin-Remodeling Protein Collaborate to Fine-Tune Circadian Transcription in Drosophila. *PLOS Genet.* **11**, e1005307 (2015).
10. Cai, Y. D. *et al.* CK2 Inhibits TIMELESS Nuclear Export and Modulates CLOCK Transcriptional Activity to Regulate Circadian Rhythms. *Curr. Biol.* **31**, 502-514.e7 (2021).
11. Chiu, J. C., Vanselow, J. T., Kramer, A. & Edery, I. The phospho-occupancy of an atypical SLIMB-binding site on PERIOD that is phosphorylated by DOUBLETIME controls the pace of the clock. *Genes Dev.* **22**, 1758–1772 (2008).
12. Cox, J. & Mann, M. MaxQuant enables high peptide identification rates, individualized p.p.b.-range mass accuracies and proteome-wide protein quantification. *Nat. Biotechnol.* **26**, 1367–1372 (2008).
13. Cox, J. *et al.* Andromeda: a peptide search engine integrated into the MaxQuant environment. *J. Proteome Res.* **10**, 1794–1805 (2011).
14. Schilling, B. *et al.* Platform-independent and label-free quantitation of proteomic data using MS1 extracted ion chromatograms in skyline: application to protein acetylation and phosphorylation. *Mol. Cell. Proteomics MCP* **11**, 202–214 (2012).
15. Lam, V. H. An Analysis of the Circadian System in Invertebrates with Characterization of Casein Kinase 1 alpha in the Regulation of the Drosophila Circadian Oscillator. By.

16. Cai, Y. D., Hidalgo Sotelo, S. I., Jackson, K. C. & Chiu, J. C. Assaying Circadian Locomotor Activity Rhythm in Drosophila. in *Circadian Clocks* (eds. Hirota, T., Hatori, M. & Panda, S.) vol. 186 63–83 (Springer US, New York, NY, 2022).

17. Schindelin, J. *et al.* Fiji: an open-source platform for biological-image analysis. *Nat. Methods* **9**, 676–682 (2012).

18. Thaben, P. F. & Westermark, P. O. Detecting Rhythms in Time Series with RAIN. *J. Biol. Rhythms* **29**, 391–400 (2014).

19. Parsons, R., Parsons, R., Garner, N., Oster, H. & Rawashdeh, O. CircaCompare: a method to estimate and statistically support differences in mesor, amplitude and phase, between circadian rhythms. *Bioinformatics* **36**, 1208–1212 (2020).

20. Wang, Q., Abruzzi, K. C., Rosbash, M. & Rio, D. C. Striking circadian neuron diversity and cycling of Drosophila alternative splicing. *eLife* **7**, e35618 (2018).

21. Wang, C. *et al.* GPS 5.0: An Update on the Prediction of Kinase-specific Phosphorylation Sites in Proteins. *Genomics Proteomics Bioinformatics* **18**, 72–80 (2020).

22. Kweon, H. K. & Andrews, P. C. Quantitative analysis of global phosphorylation changes with high-resolution tandem mass spectrometry and stable isotopic labeling. *Methods San Diego Calif* **61**, 251–259 (2013).

23. Lee, E. *et al.* Phosphorylation of a Central Clock Transcription Factor Is Required for Thermal but Not Photic Entrainment. *PLoS Genet.* **10**, e1004545 (2014).
